# Supplementary material for: Epidermal electronic-tattoo for plant immune response monitoring
Source: Nat Commun. 2025 Apr 4;16:3244. doi: 10.1038/s41467-025-58584-x (PMC11971386; doi:10.1038/s41467-025-58584-x)
Supplement: Supplementary file 1 — Supplementary Information [file 41467_2025_58584_MOESM1_ESM.pdf]

Epidermal electronic-tattoo for plant immune response monitoring

He *et al.*

Supplementary Table 1. Comparison of recent plant-attached electrodes.

**Reference**	**1**	**2, 3**	**4**	**5**	**6**	**This work**
**Substrate**	Polyimide	N/A	Adhesive Tape	PVA fiber film	PLA fiber film	N/A
**Electrode material**	Ti and Au	PProDOT-Cl	Ni	Liquid Metal	CNT	AgNWs
**Thickness**	350 μm	1 μm	21-42 μm (excluding the substrate)	9-12 μm (excluding the substrate)	~2 um	100 nm
**Attachment Method**	Insertion into leaves	Vapor coating on the leaf surface	Fixed by adhesive tape	Dissolved PVA serving as an adhesive to adhere to the leaf surface	In-situ electrospinning and spray coating	In-water transfer printing
**Versatility to Multiple Plants**	Limited (induces wounding in leaves)	Limited (requires exposer to a 100-mtorr vacuum in a reactor for 20 min)	Limited (can cause damage to the delicate leaf tissues when applied or removed)	Moderate (limited flexibility may cause partial detachment from the leaf surface as it dries)	Limited (faces challenges with delicate surfaces and uniform application across plant species)	Good (The in-water transfer printing process is safe, gentle, and suitable for delicate surfaces, showing no harm to living plants like *A. thaliana*)
**Conformability**	Limited (The microneedle patch is relatively thick and lacks flexibility)	Excellent (The vapor-printed polymer electrodes conform well to leaf surfaces)	Limited (The Ni film-based electrodes are primarily restricted to smooth surfaces)	Good (The liquid-metal-based electrodes exhibit good conformability and adhesion to smooth leaf surfaces)	Good (The CNT/PLA electrodes demonstrate good conformability on smooth leaf surfaces)	Excellent (The substrate-free and ultrathin plant e-tattoo demonstrates exceptional conformability to various leaf surfaces, including those with hair)
**Optical Transparency**	Adequate	Poor	Poor	Poor	Poor	Good
**Continuous Monitoring**	Yes	No	No	No	Yes	Yes
**Applications**	Leaf water content monitoring	deep tissue damage caused by dehydration, ultraviolet A and ozone exposure	Leaf water loss monitoring	Leaf water content monitoring	Drought stress detection	Plant immune response monitoring

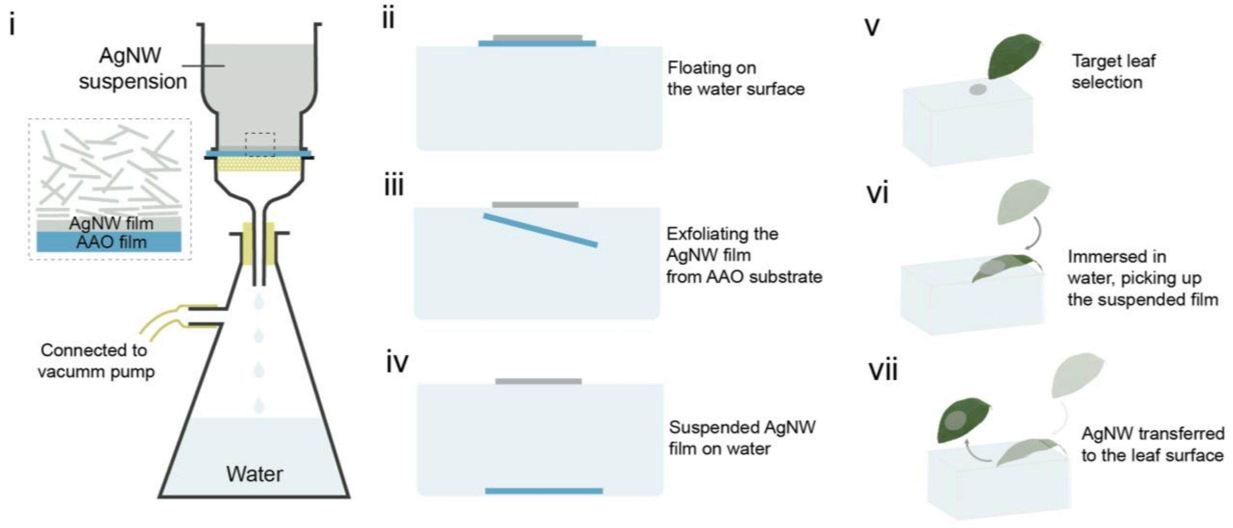

Supplementary Fig. 1. Fabrication process and in-water transfer printing of the plant e-tattoo.

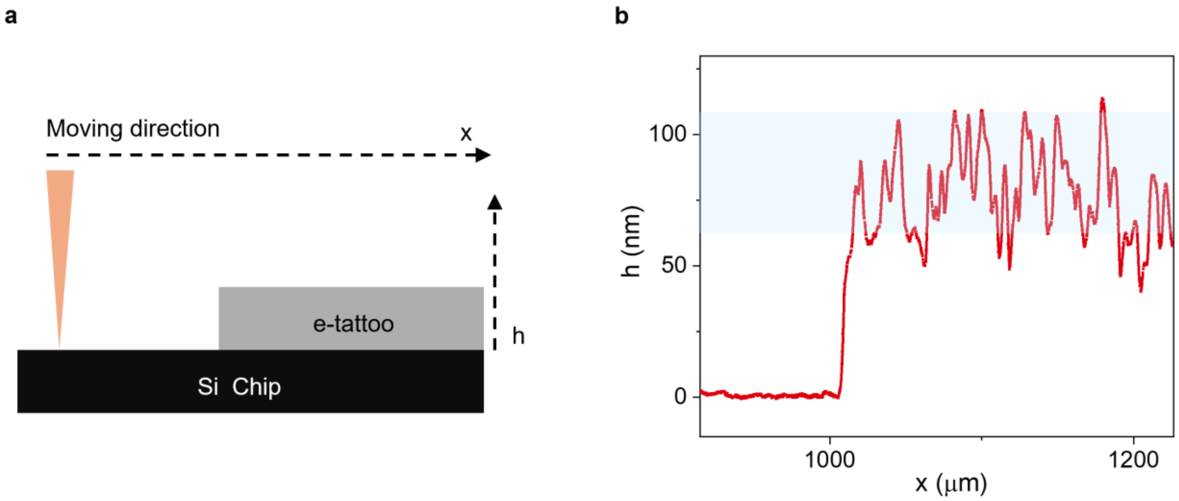

Supplementary Fig. 2. Typical step-height profile (film thickness) of the plant e-tattoo. a, Illustration of thickness measurement along the scanning direction. The e-tattoo was transferred onto an amorphous silicon chip, and the surface profile at the e-tattoo edge was measured. **b,** Result of the surface profile measurement. Source data are provided as a Source Data file.

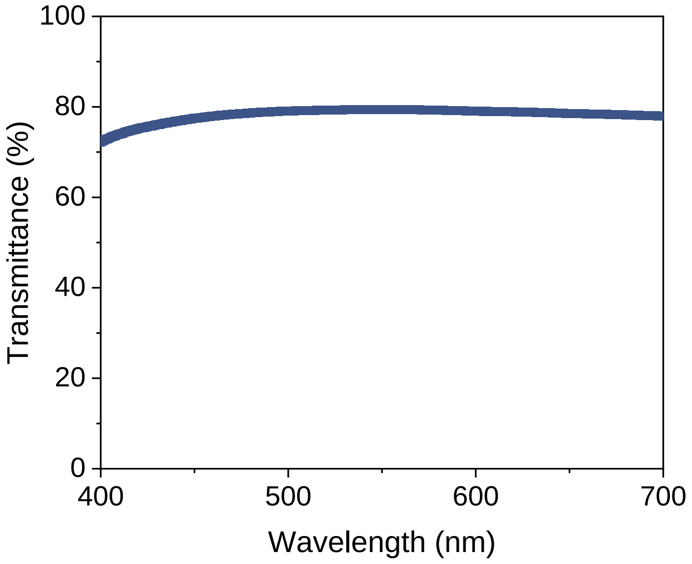

Supplementary Fig. 3. Transmission spectrum of the AgNW film. Source data are provided as a Source Data file.

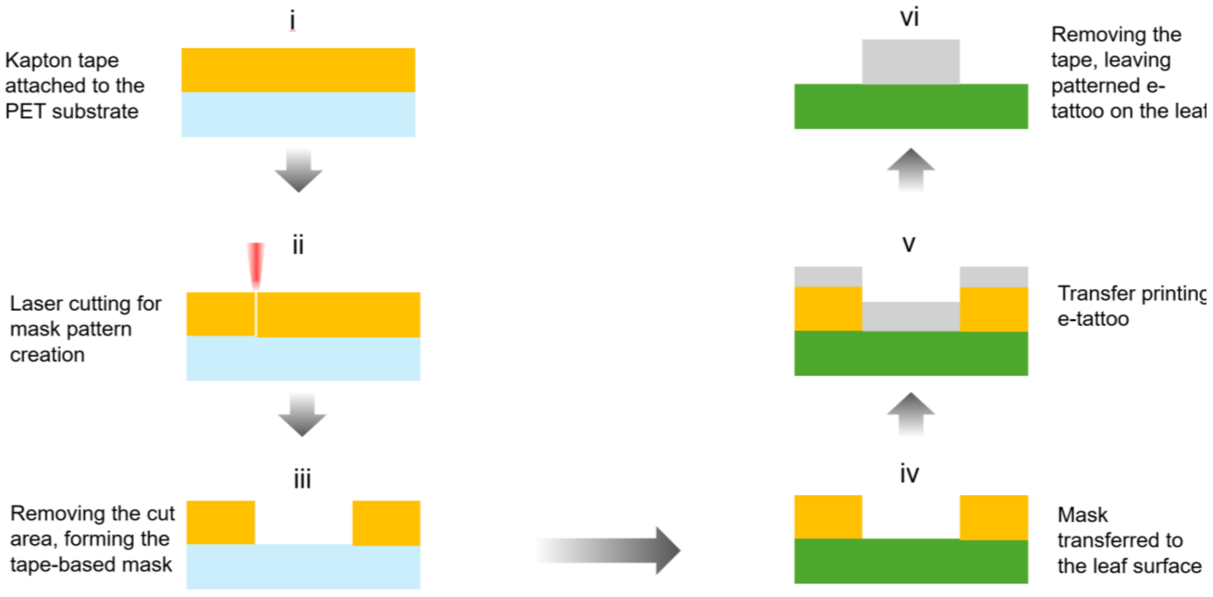

Supplementary Fig. 4. Procedure for creating designed patterns with the plant e-tattoo.

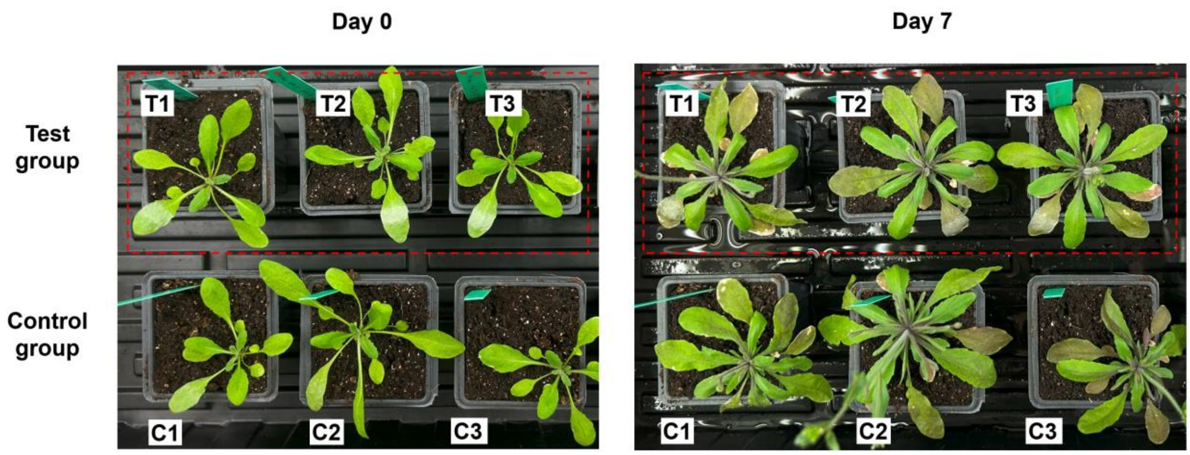

Supplementary Fig. 5. Biocompatibility experiment with *A. thaliana* plants. In the test group, plant e-tattoos were applied to the 5th to 7th leaves, while the control group remained untreated. After 7 days under identical cultivation conditions, both groups exhibited normal growth, with comparable increases in leaf size and number. The older leaves in both groups displayed a darker green coloration, which is a typical phenomenon in plants.

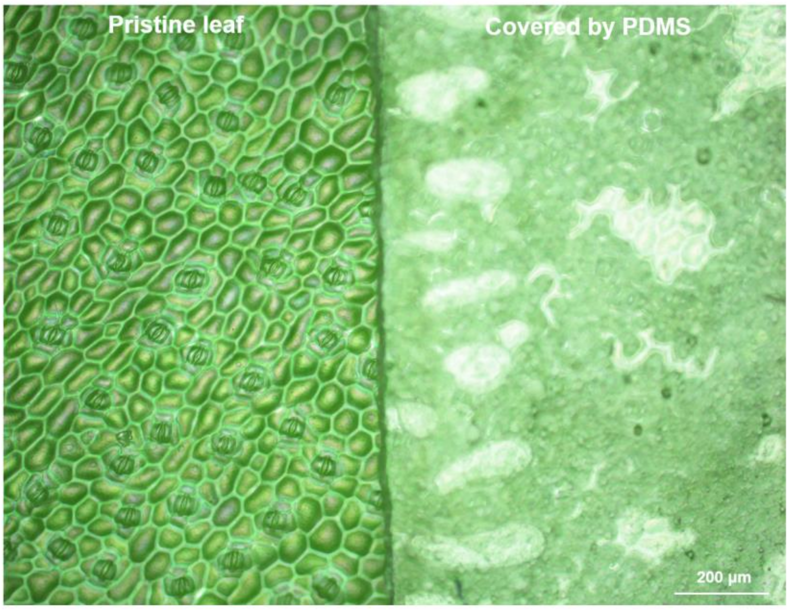

Supplementary Fig. 6. OM image of the abaxial leaf surface partially covered by PDMS. The experiment was repeated three times with similar results.

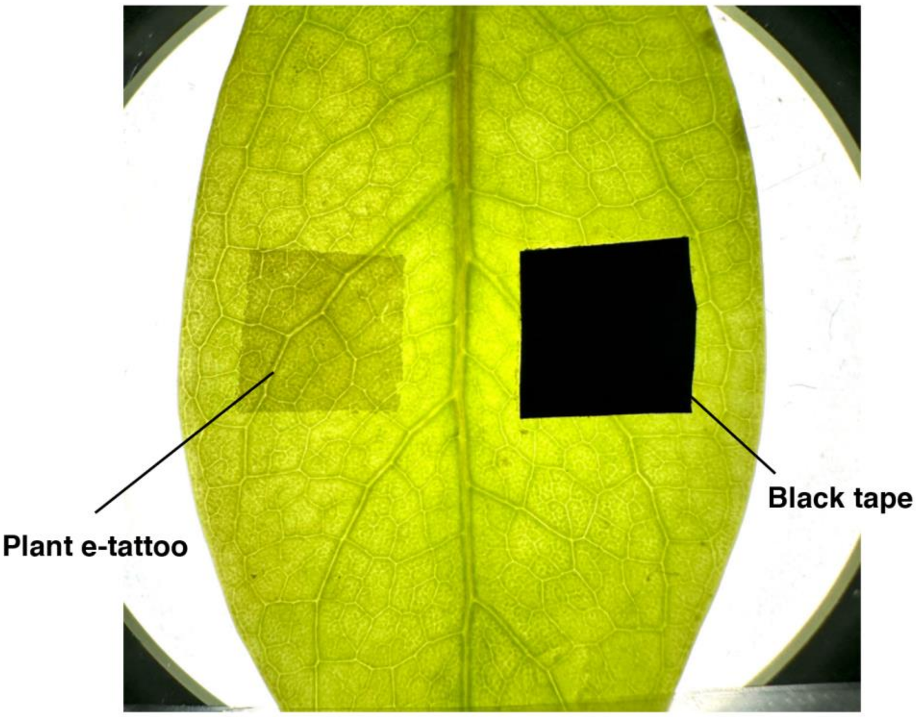

Supplementary Fig. 7. Photo of a leaf with e-tattoo and black tape attached. A light source placed behind the leaf highlights the difference in light transmission: the black tape blocks the light, while the e-tattoo allows light to pass through, making the veins within the leaf tissue visible.

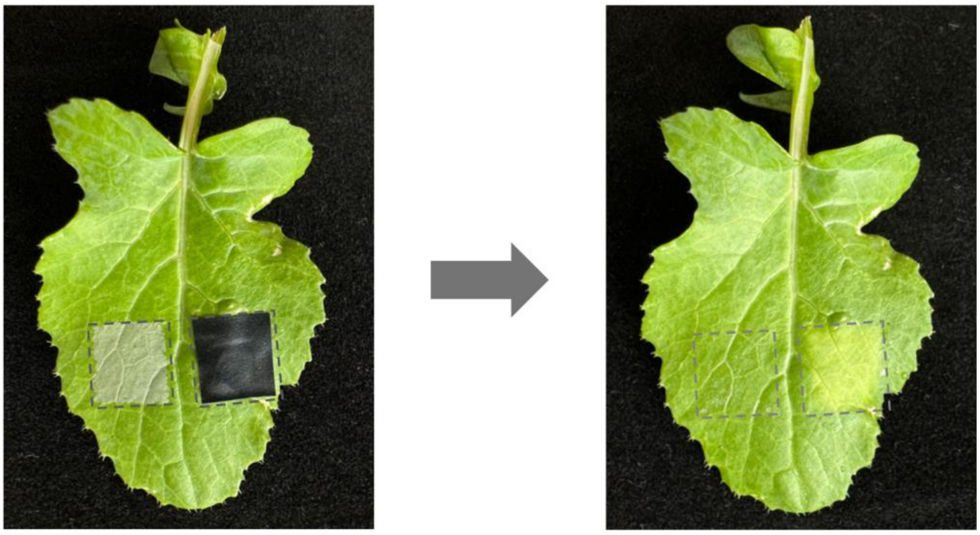

Supplementary Fig. 8. Images of a *Brassica rapa* (oilseed sarson) leaf with the e-tattoo and black tape attached and subsequently removed after two-week attachment.

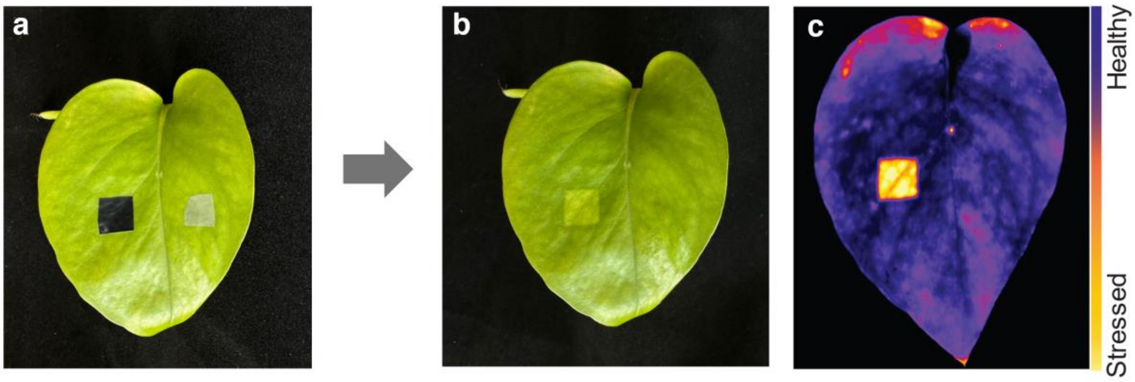

Supplementary Fig. 9. Long-term attachment effects on leaf health. a, Image of a pothos leaf with the e-tattoo and black tape attached. **b**, Image of a pothos leaf after the removal of e-tattoo and black tape after three-week attachment. **c**. Fluorescence image of the pothos leaf after removing the e-tattoo and black tape, showing the black tape-covered area (left) and the e-tattoo-covered area (right).

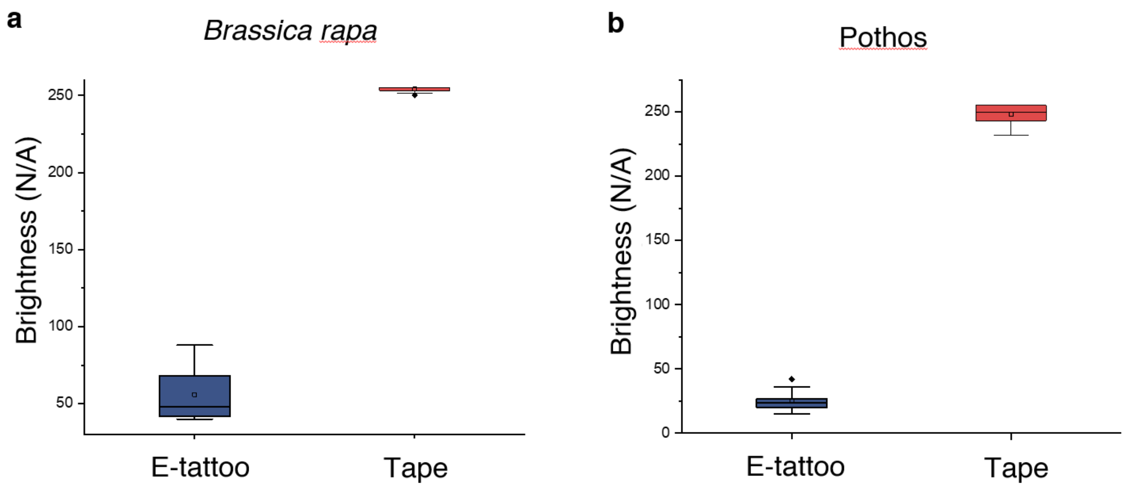

Supplementary Fig. 10. Comparison of the impact of the e-tattoo and black tape on leaf tissue health. Brightness values of individual pixels were quantified by adjusting the intensity range of the entire image to 55–255. The brightness intensities of the areas covered by the e-tattoo and black tape were analyzed using box plots for **a**, *Brassica rapa* leaf, and **b**, Pothos leaf. In the box plots, the horizontal line indicates the median, the box boundaries represent the 25th and 75th percentiles, and the whiskers extend to the minimum and maximum values within 1.5 times the interquartile range (IQR). Data points beyond this range are considered outliers and are shown as individual markers. The box plots are based on $n = 10$ data points for each group. Statistical significance between groups was assessed using one-way ANOVA, with $p < 0.05$ considered significant. Source data are provided as a Source Data file.

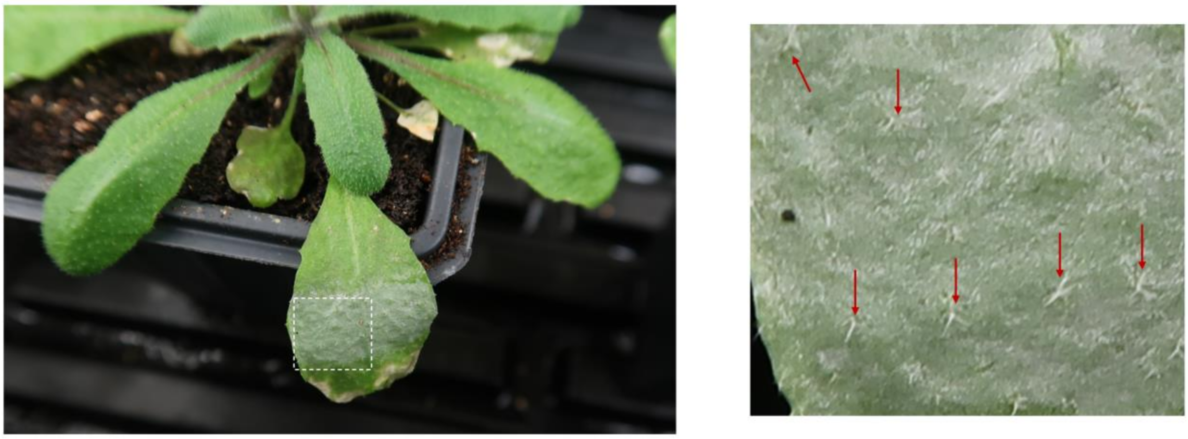

Supplementary Fig. 11. Images of the e-tattoo on the *A. thaliana* leaf. Red arrows highlight the presence of trichomes on the leaf surface.

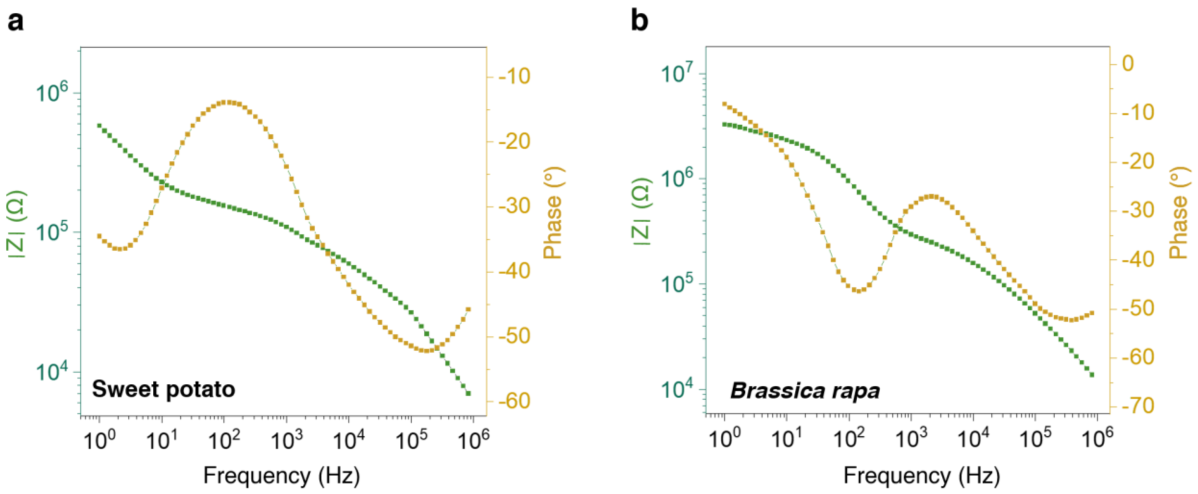

Supplementary Fig. 12. EIS data collected on leaves from crop species. a-b, Bode plots acquired with e-tattoo on the leaf of sweet potato and *Brassica rapa*, respectively. Source data are provided as a Source Data file.

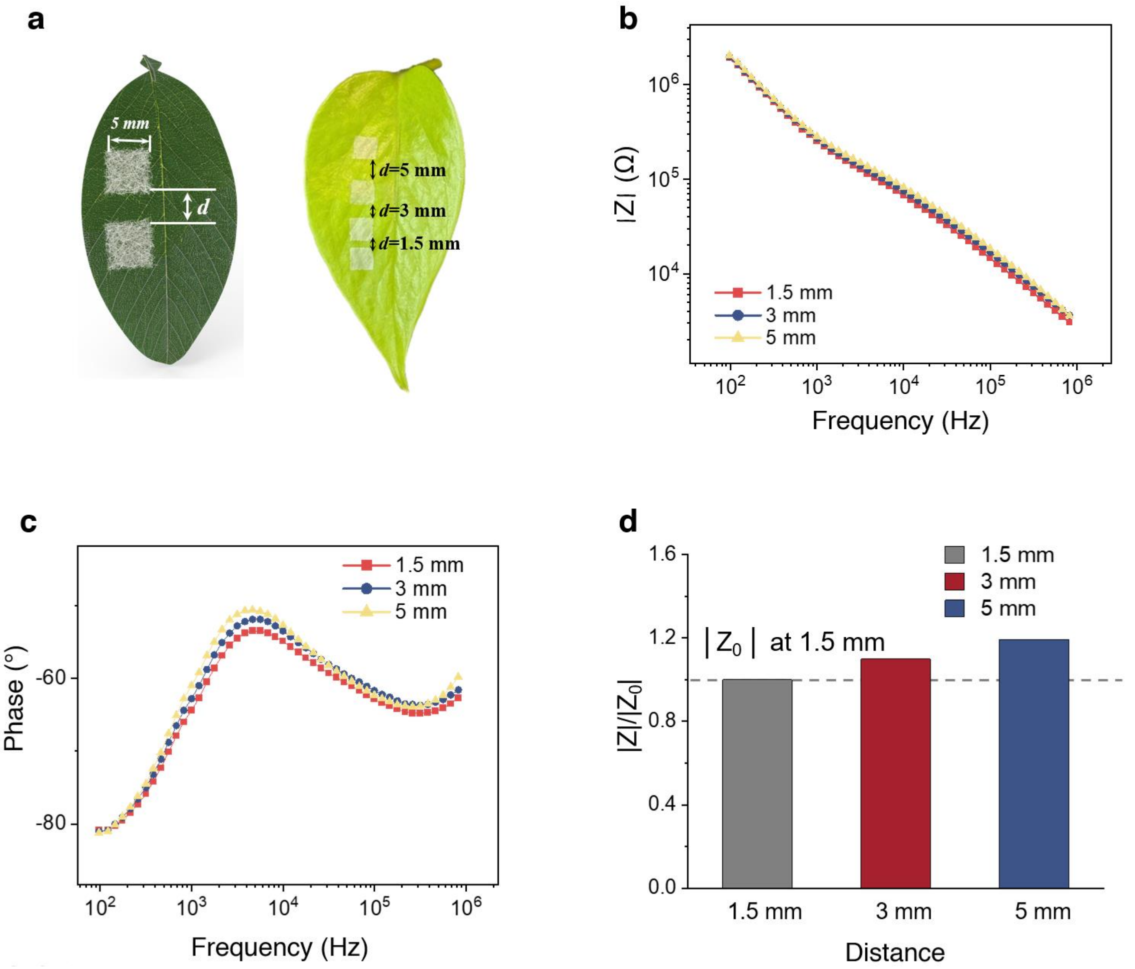

Supplementary Fig. 13. Effect of electrode spacing on leaf impedance measurements. a, Schematic showing different electrode spacing distances (d = 1.5 mm, 3 mm, and 5 mm) on leaves. **b**, Impedance magnitude spectra of leaf measured at different electrode spacings. **c**, Phase spectra of leaf at different electrode spacings. **d**, Relative impedance magnitude at 5 kHz of the leaf measured with e-tattoo electrodes at distances of 1.5 mm, 3 mm, and 5 mm, normalized to the impedance at a 1.5 mm distance. Source data are provided as a Source Data file.

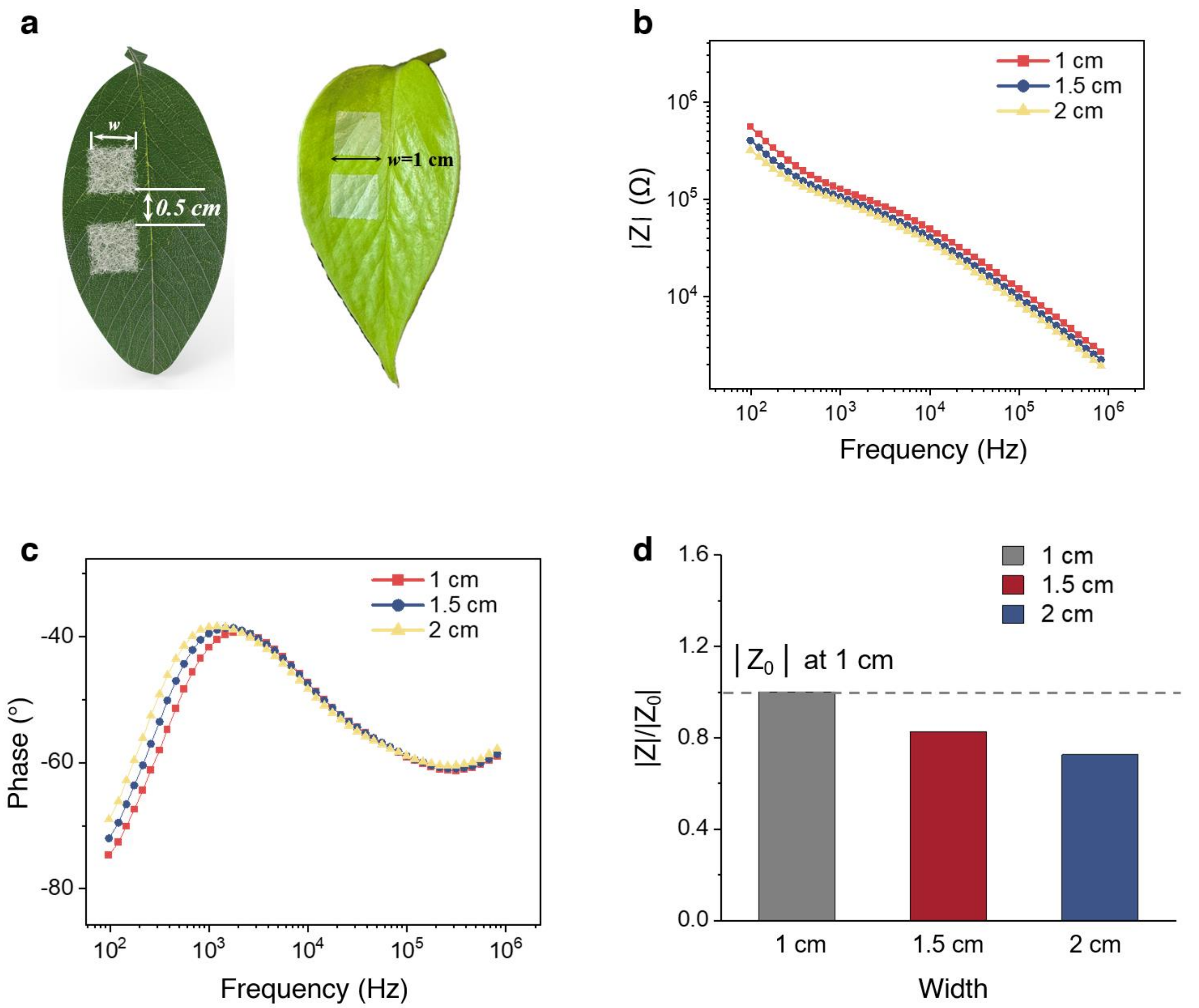

Supplementary Fig. 14. Effect of electrode width on leaf impedance measurements. a, Schematic and photograph illustrating the arrangement of e-tattoo electrodes; **b**. Impedance magnitude spectra obtained using e-tattoo electrodes with widths of 1 cm, 1.5 cm, and 2 cm. **c**, Phase spectra recorded with e-tattoo electrodes at widths of 1 cm, 1.5 cm, and 2 cm. **d**. Relative impedance magnitude at 5 kHz of the leaf measured with e-tattoo electrodes at widths of 1 cm, 1.5 cm, and 2 cm, normalized to the impedance with a 1 cm width. Source data are provided as a Source Data file.

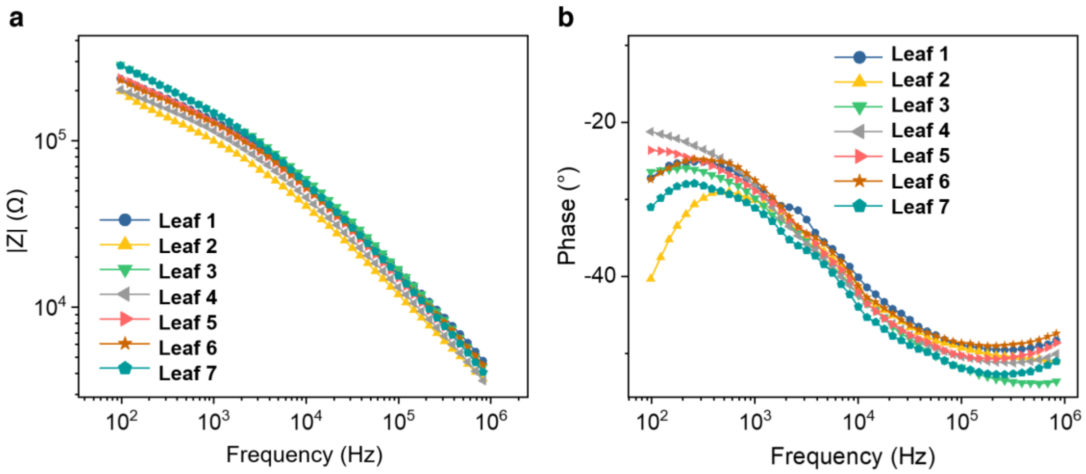

Supplementary Fig. 15. EIS of *A. thaliana* leaves with different sizes and growth stages. a, Impedance magnitude spectra obtained from different leaves. **b**, Phase spectra obtained from different leaves. Source data are provided as a Source Data file.

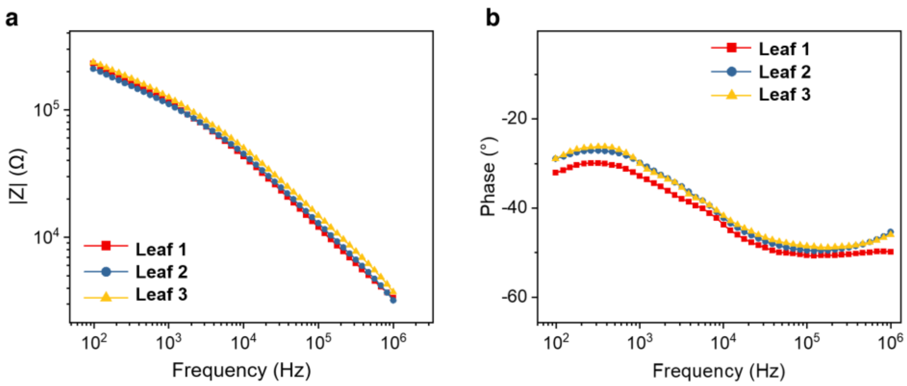

Supplementary Fig. 16. EIS of different leaves on the same *A. thaliana* plant. a, Impedance magnitude spectra obtained from different leaves. **b**, Phase spectra obtained from different leaves. Source data are provided as a Source Data file.

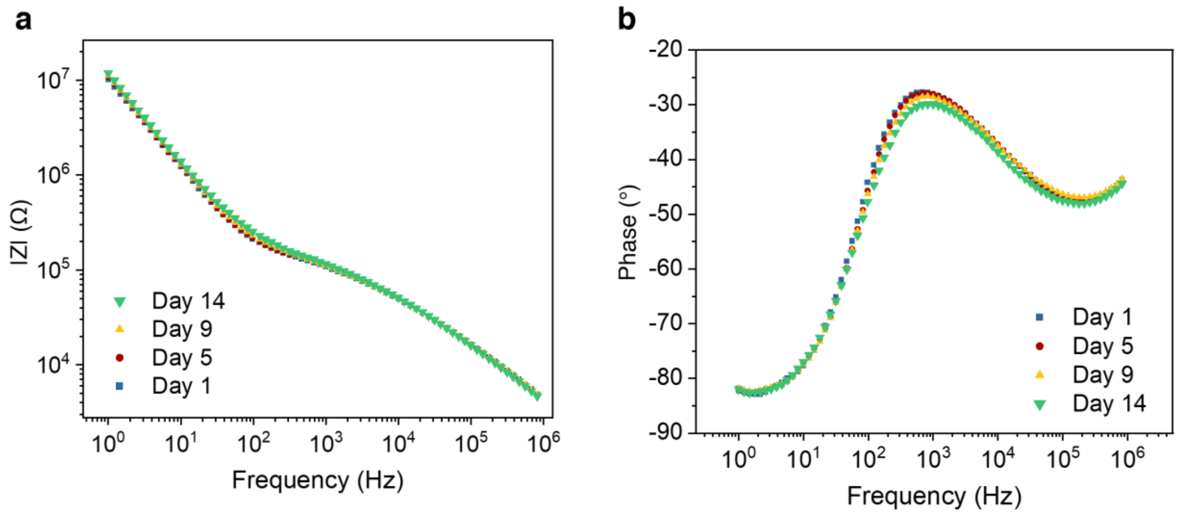

Supplementary Fig. 17. EIS data collected on an intact *A. thaliana* plant over a 14-day period. a, Impedance magnitude spectra in different days. **b,** Phase spectra in different days. Source data are provided as a Source Data file.

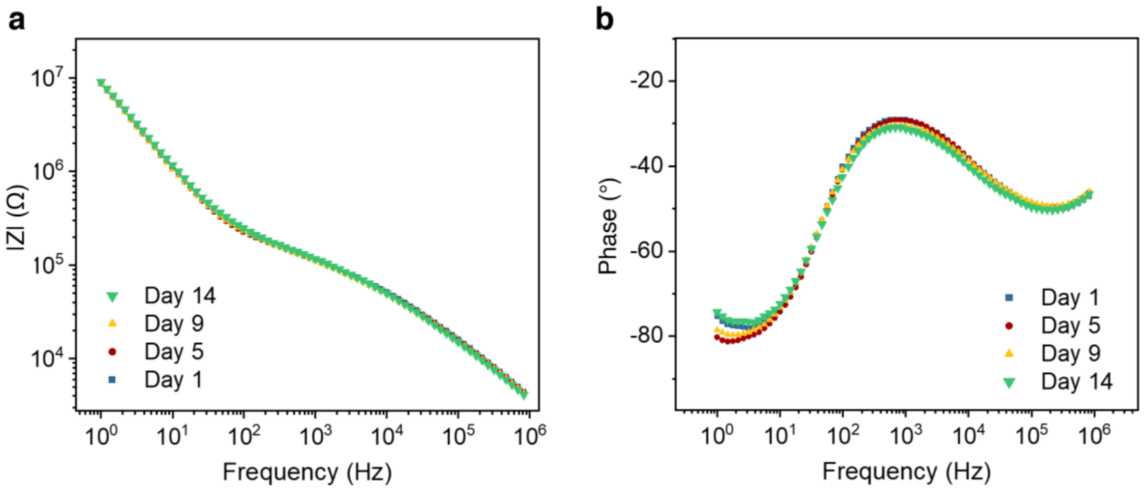

Supplementary Fig. 18. EIS data collected on another intact *A. thaliana* plant over a 14-day period. **a,** Impedance magnitude spectra in different days. **b,** Phase spectra in different days. Source data are provided as a Source Data file.

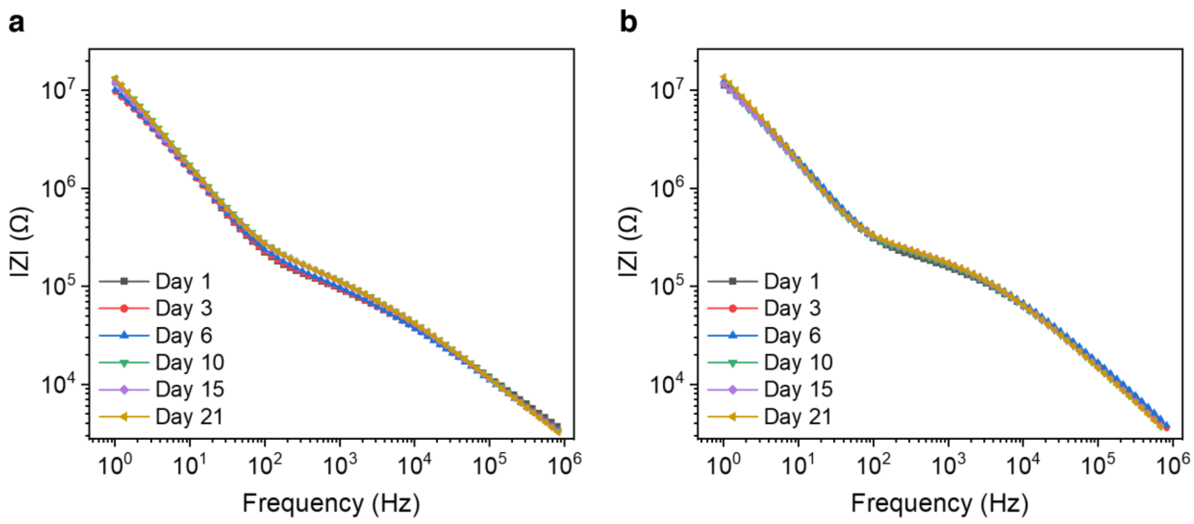

Supplementary Fig. 19. EIS data collected on two intact *A. thaliana* plants over a 21-day period. **a,** Impedance magnitude spectra in different days of a *A. thaliana* plant. **b,** Impedance magnitude spectra in different days of another *A. thaliana* plant. Source data are provided as a Source Data file.

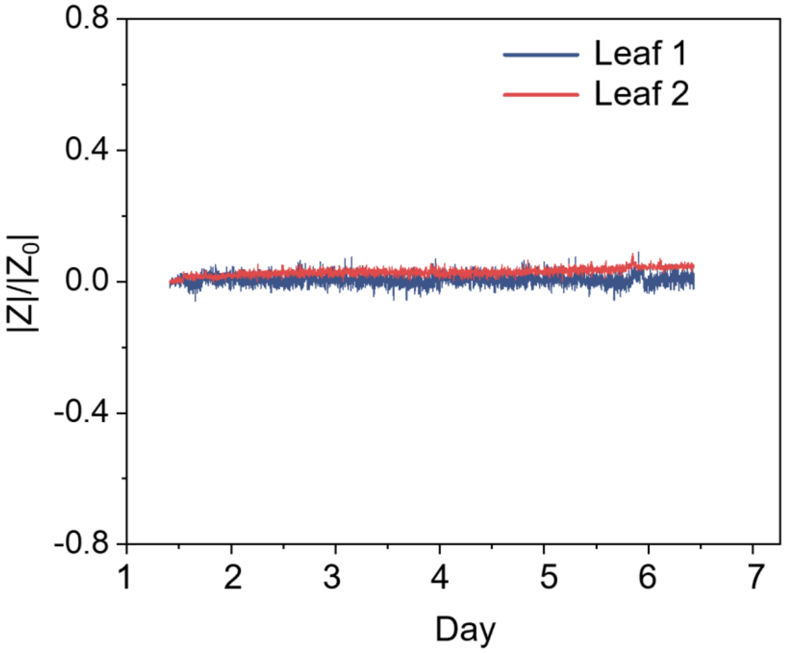

Supplementary Fig. 20. Continuous impedance monitoring of two leaves on intact *A. thaliana* plants for 5 days. Source data are provided as a Source Data file.

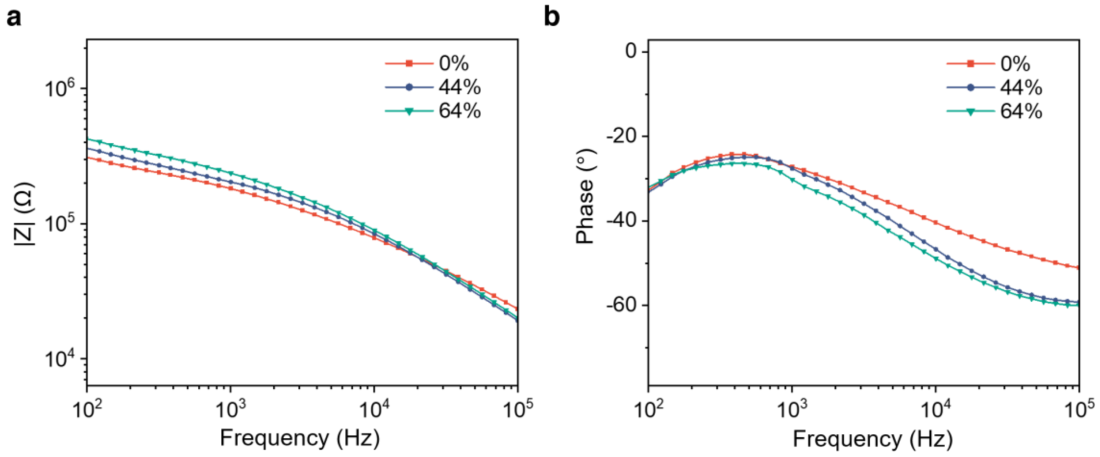

Supplementary Fig. 21. EIS response of plant leaves to dehydration. a, Impedance magnitude spectra of a *A. thaliana* leaf at different levels of LWC (loss of water content)**. b**, Phase spectra of a *A. thaliana* leaf at different levels of LWC. Source data are provided as a Source Data file.

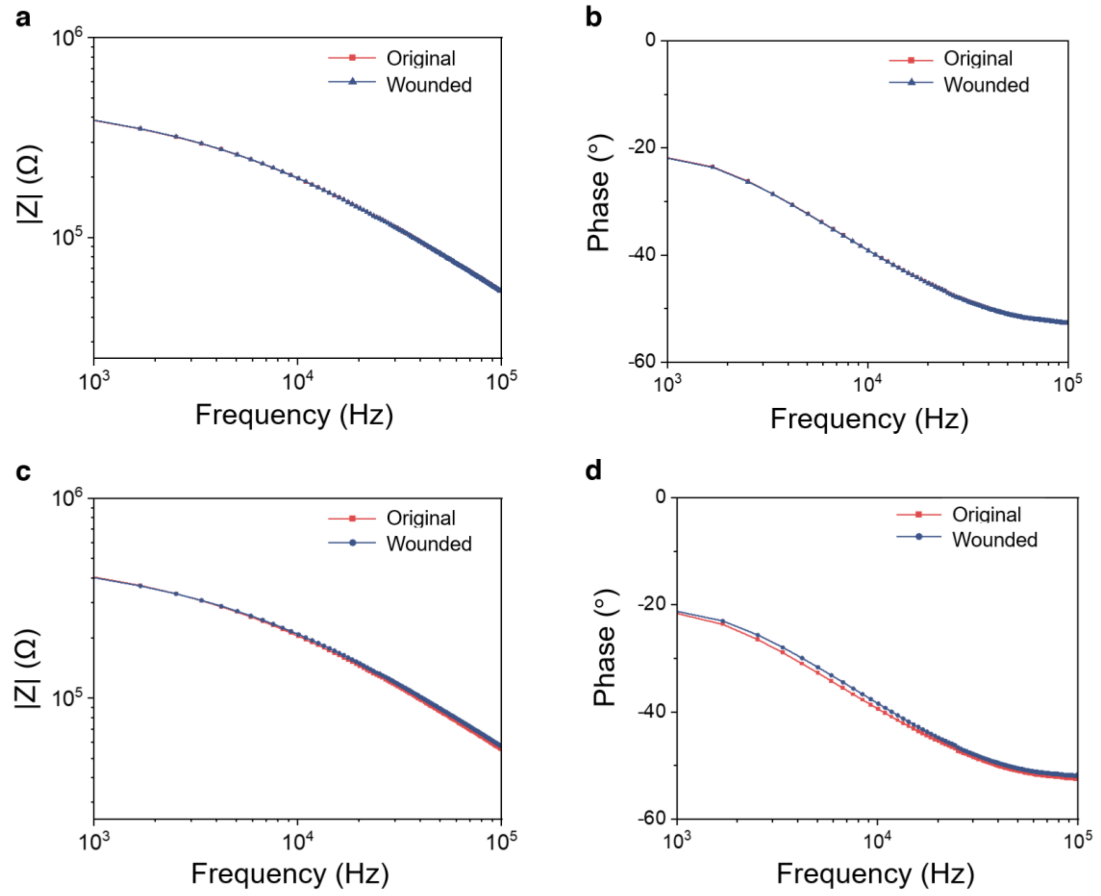

Supplementary Fig. 22. EIS response of plant leaves to wounding. The wound was created by making a small hole with a tweezer at the midpoint between the two e-tattoo electrodes. EIS measurements were taken 10 minutes post-wounding to assess the response. **a-b**, Impedance magnitude and phase spectra of a sweet potato leaf before and after wounding. **c-d**, Impedance magnitude and phase spectra of another sweet potato leaf before and after wounding with two cuts. Source data are provided as a Source Data file.

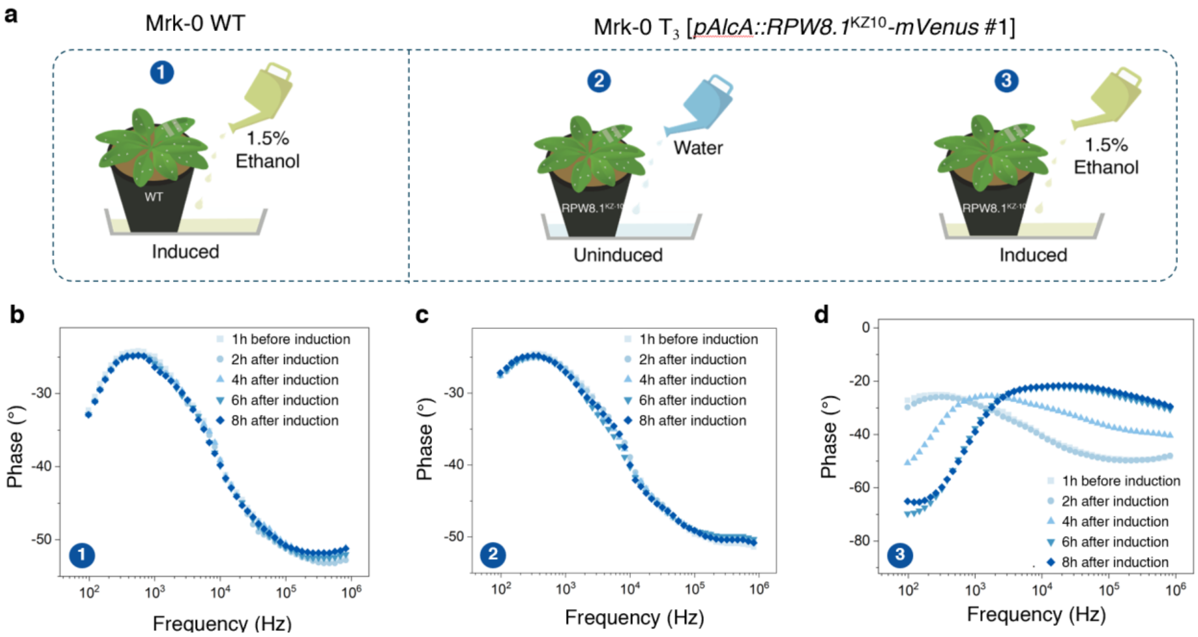

Supplementary Fig. 23. Phase spectra of the test and control group *A. thaliana* plants. a, Experimental setup schematics for the control and test groups. **b-d,** Phase spectra overtime of the test and control group plants. Source data are provided as a Source Data file.

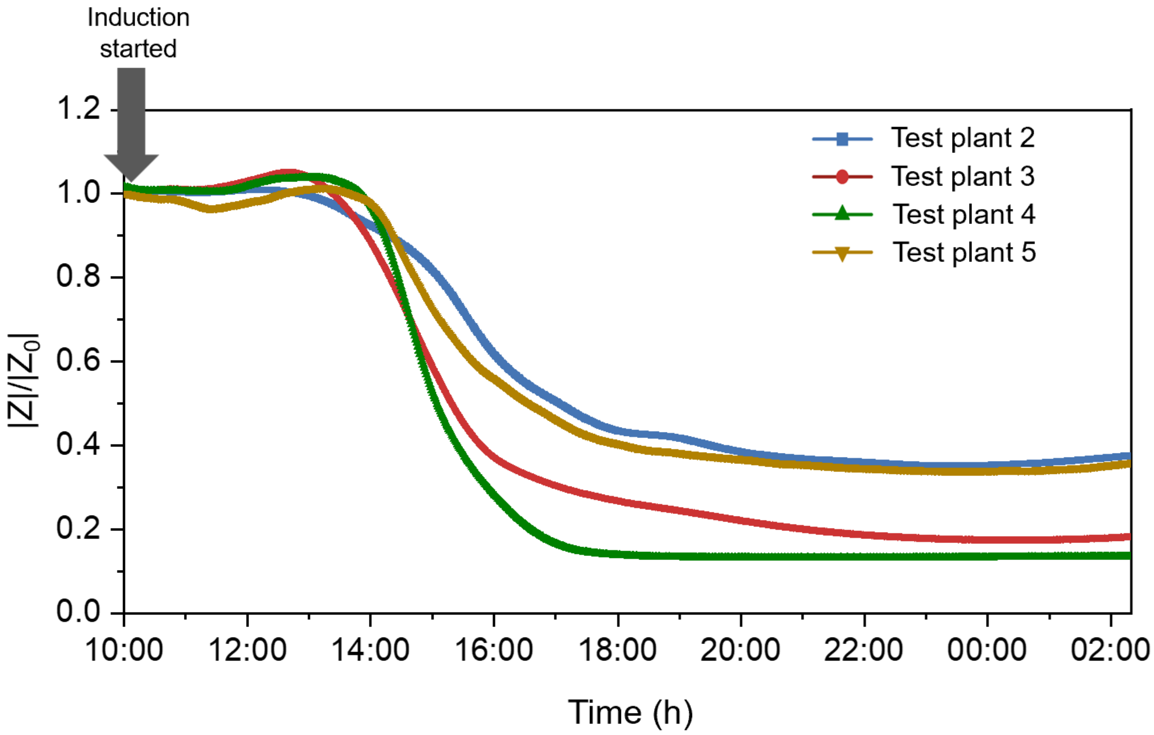

Supplementary Fig. 24. Normalized impedance magnitude at 2 kHz of the test group over time post induction with ethanol. The test plants here (test plant 2-5) are from the same batch of the ones in Fig. 5. Source data are provided as a Source Data file.

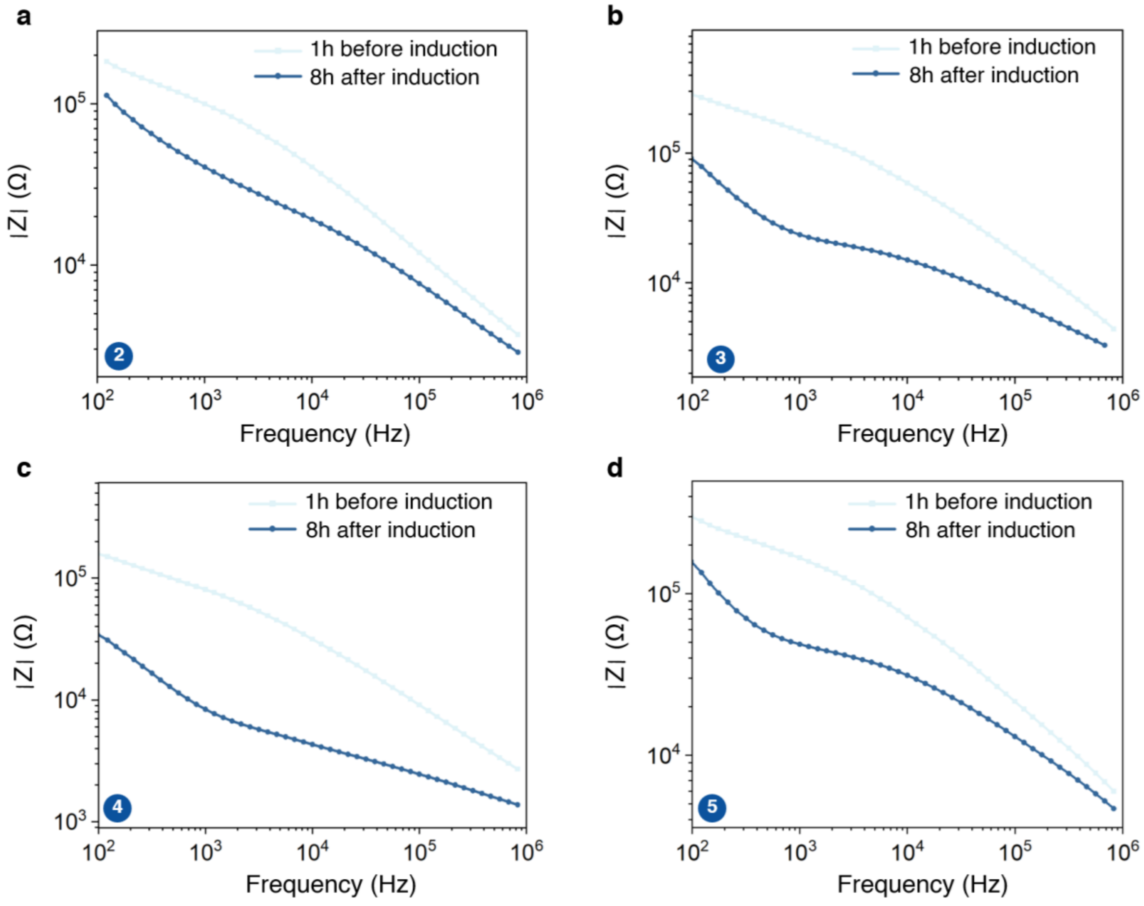

Supplementary Fig. 25. Impedance magnitude spectra of four different plants before and after induction with ethanol. a, Plant 2, **b**, Plant 3, **c**, Plant 4, **d**, Plant 5. Source data are provided as a Source Data file.

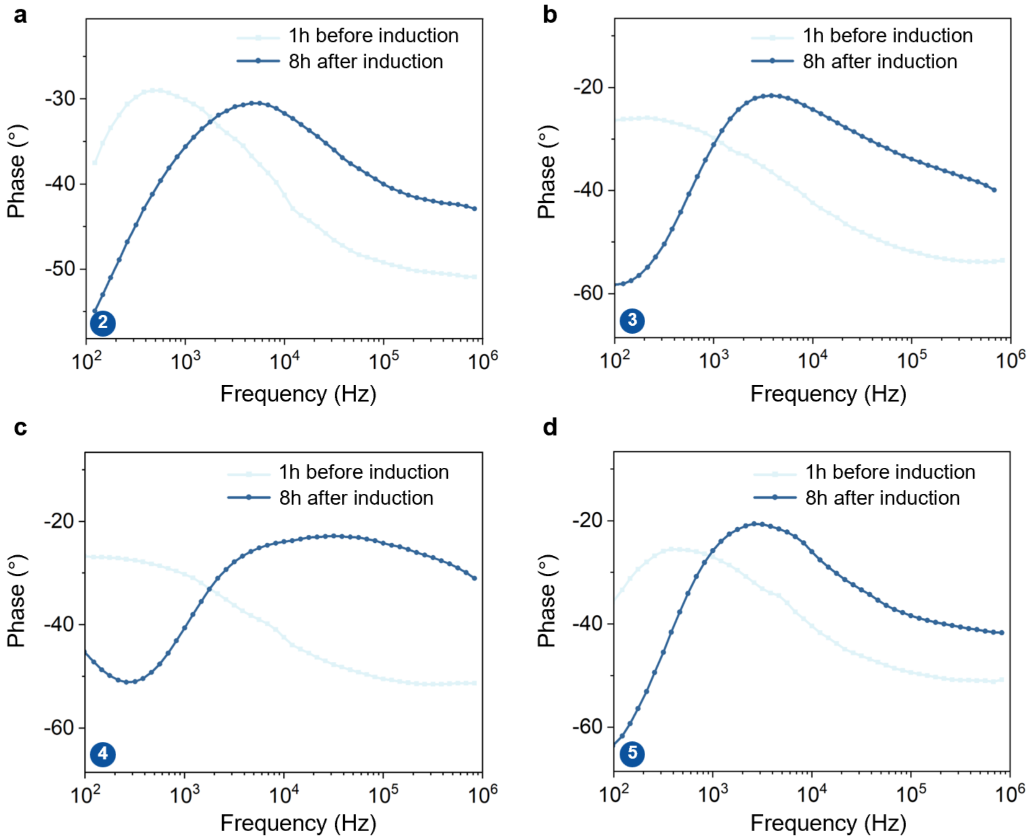

Supplementary Fig. 26. Phase spectra of four different plants before and after induction with ethanol. a, Plant 2, **b**, Plant 3, **c**, Plant 4, **d**, Plant 5. Source data are provided as a Source Data file.

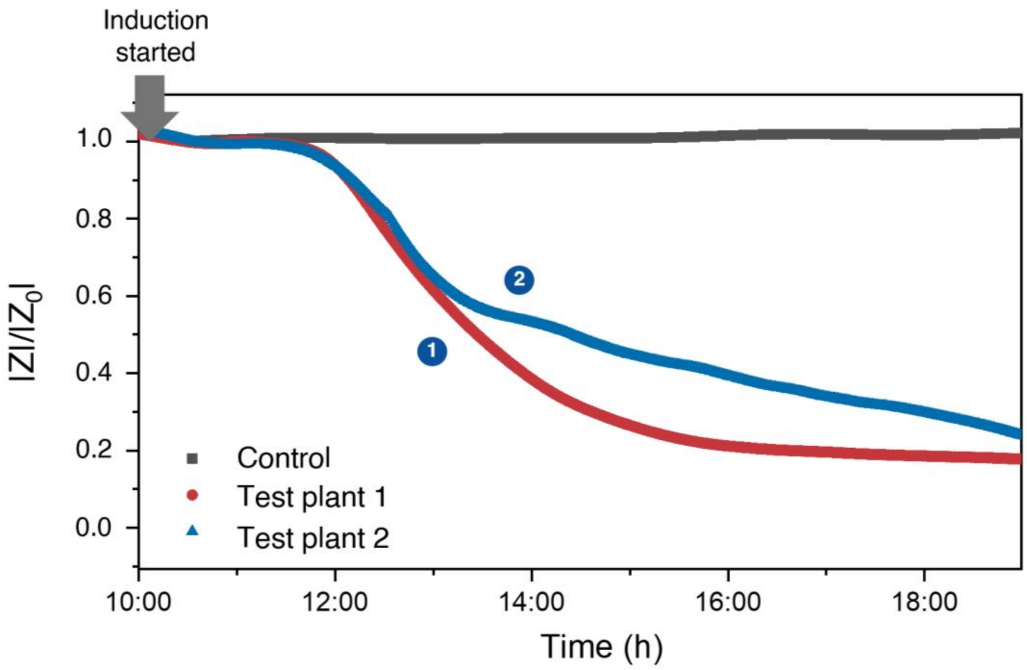

Supplementary Fig. 27. Normalized impedance magnitude at 2 kHz of the test group and control group over time post induction with ethanol or water. This experiment is done with a different batch of *A. thaliana* Mrk-0 plants with inducible *DM7* transgene (T_3[*pAlcA::RPW8.1*^{KZ10}-*mVenus* #1]). The experimental set up is consistent with that discussed in Fig. 5. The control group consists of Mrk-0 plants treated with pure water, while the test group was induced with 1.5% ethanol. Source data are provided as a Source Data file.

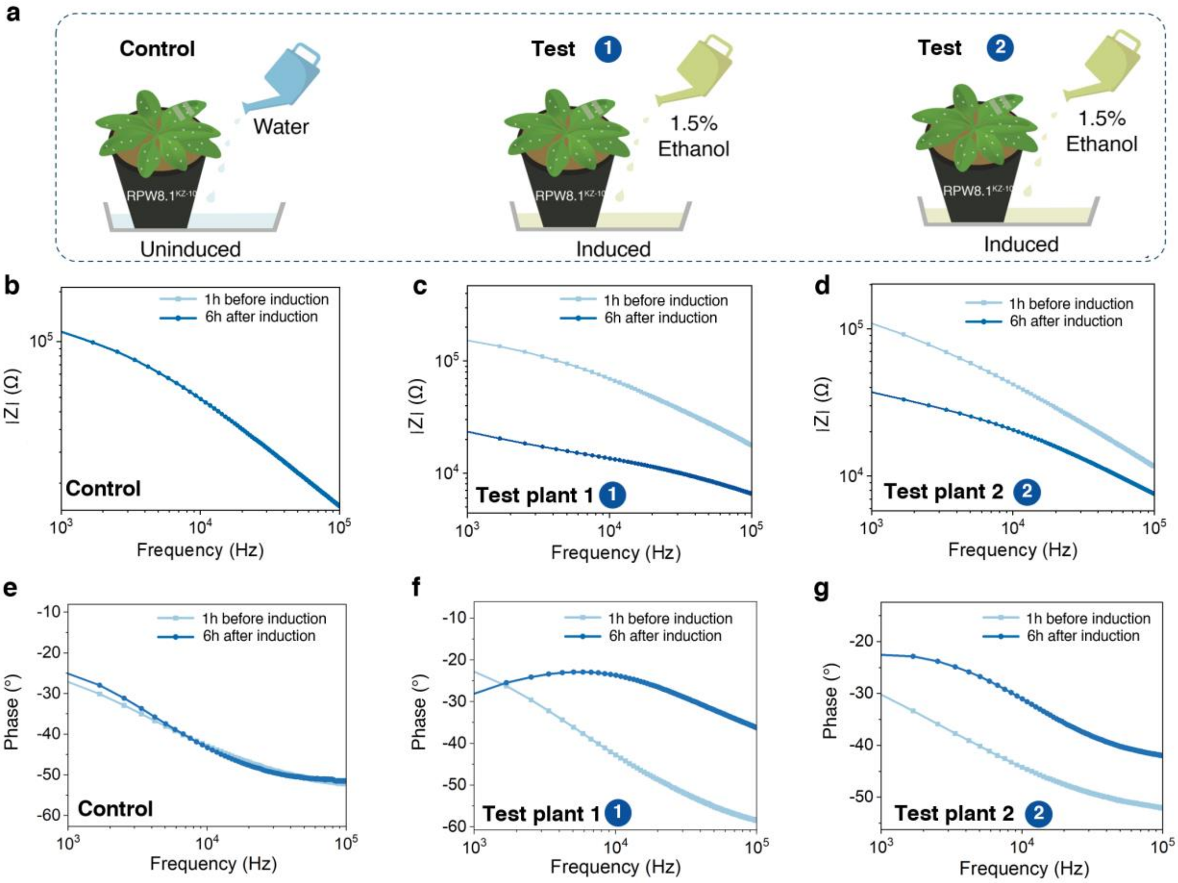

Supplementary Fig. 28. *A. thaliana* autoimmune response monitoring with plant e-tattoo. a, Experimental setup schematics for the control and test groups. **b-d,** Impedance magnitude spectra of the control and test groups before and after induction. **e-f**, Phase spectra of the control and test groups before and after induction. Source data are provided as a Source Data file.

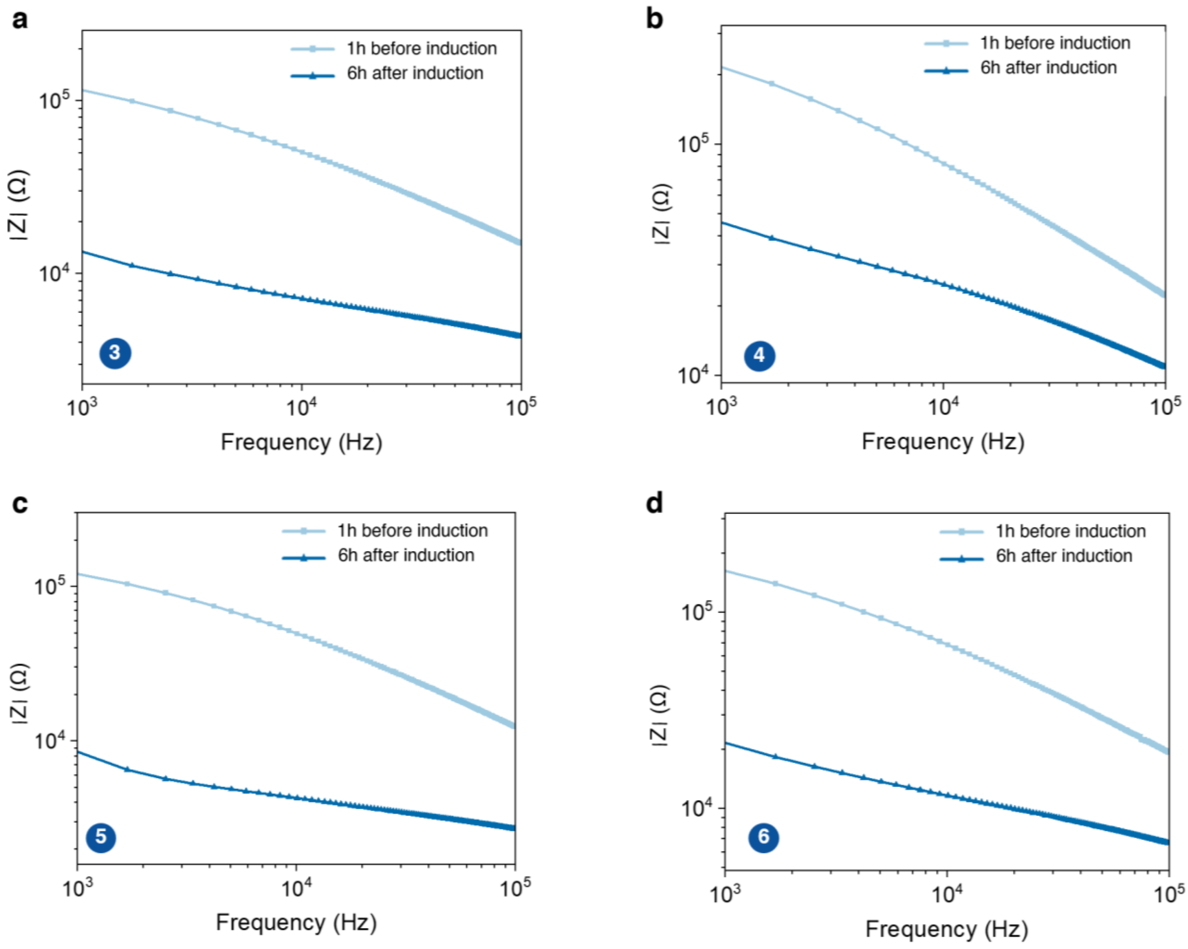

Supplementary Fig. 29. Impedance magnitude spectra of four different plants before and after induction with ethanol. a, Plant 3, **b,** Plant 4, **c,** Plant 5, **d**, Plant 6. Source data are provided as a Source Data file.

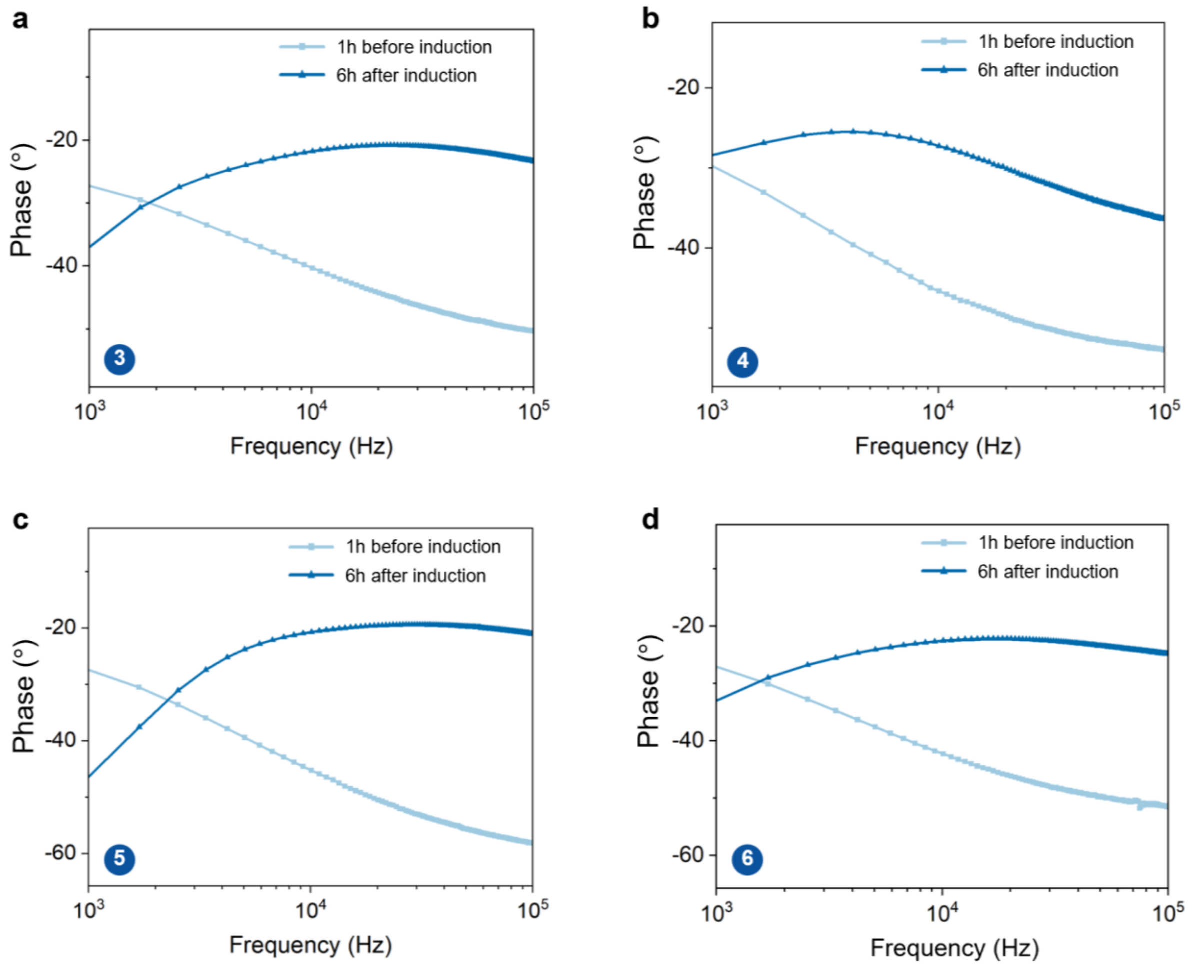

Supplementary Fig. 30. Phase spectra of four different plants before and after induction with ethanol. a, Plant 3, **b**, Plant 4, **c**, Plant 5, **d**, Plant 6. Source data are provided as a Source Data file.

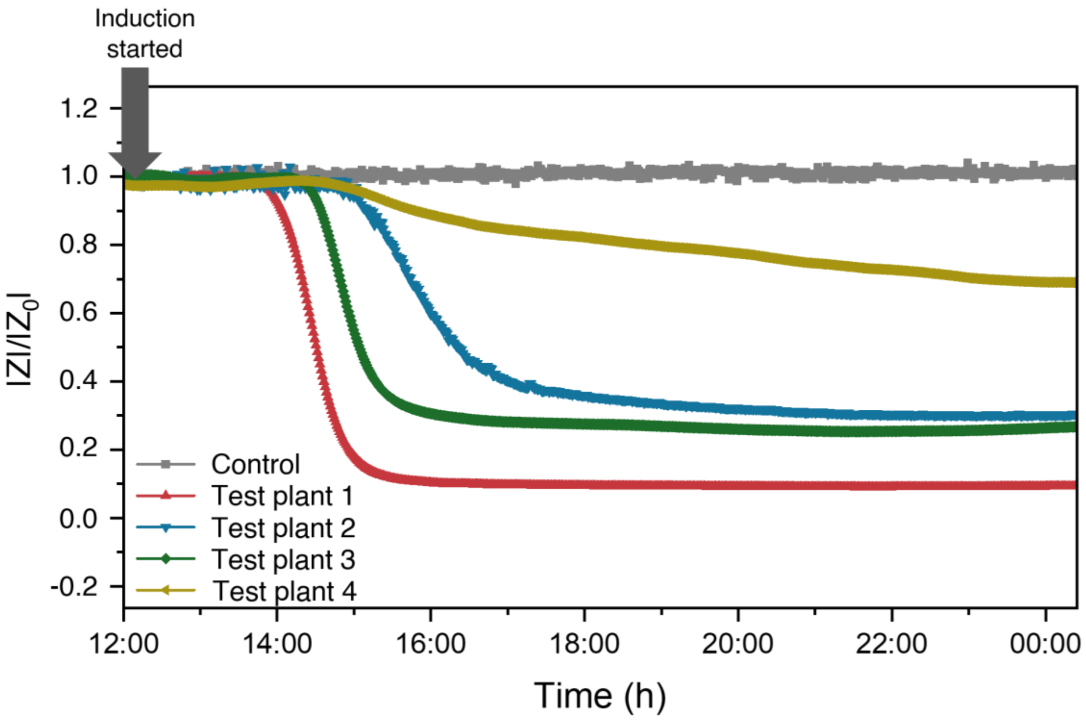

Supplementary Fig. 31. Normalized impedance magnitude at 2 kHz of the test and control groups over time post induction with ethanol or water. This experiment is done with a different batch of *A. thaliana* Mrk-0 plants with inducible *DM7* transgene (Mrk-0 T_3[*pAlcA::RPW8.1*^{KZ10}-*mVenus* #1]). The experimental set up is consistent with previous two. The control group consists of Mrk-0 plants treated with pure water, while the test group was induced with 1.5% ethanol. Source data are provided as a Source Data file.

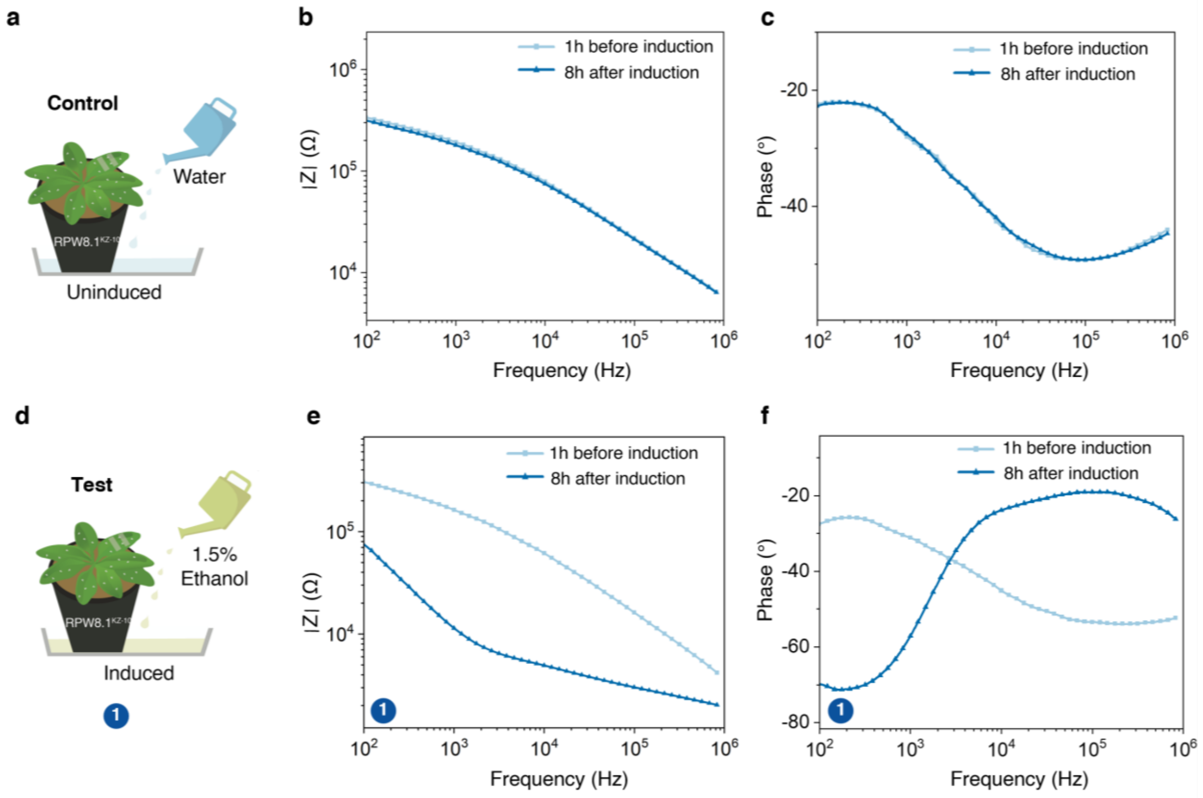

Supplementary Fig. 32. ***A. thaliana*** **autoimmune response monitoring with plant e-tattoo. a,** Experimental setup schematics for the control groups. **b-c**, Impedance magnitude spectra and phase spectra of the control plants before and after induction. **d,** Experimental setup schematics for the test groups. **e-f**, Impedance magnitude spectra and phase spectra of the test plants before and after induction. Source data are provided as a Source Data file.

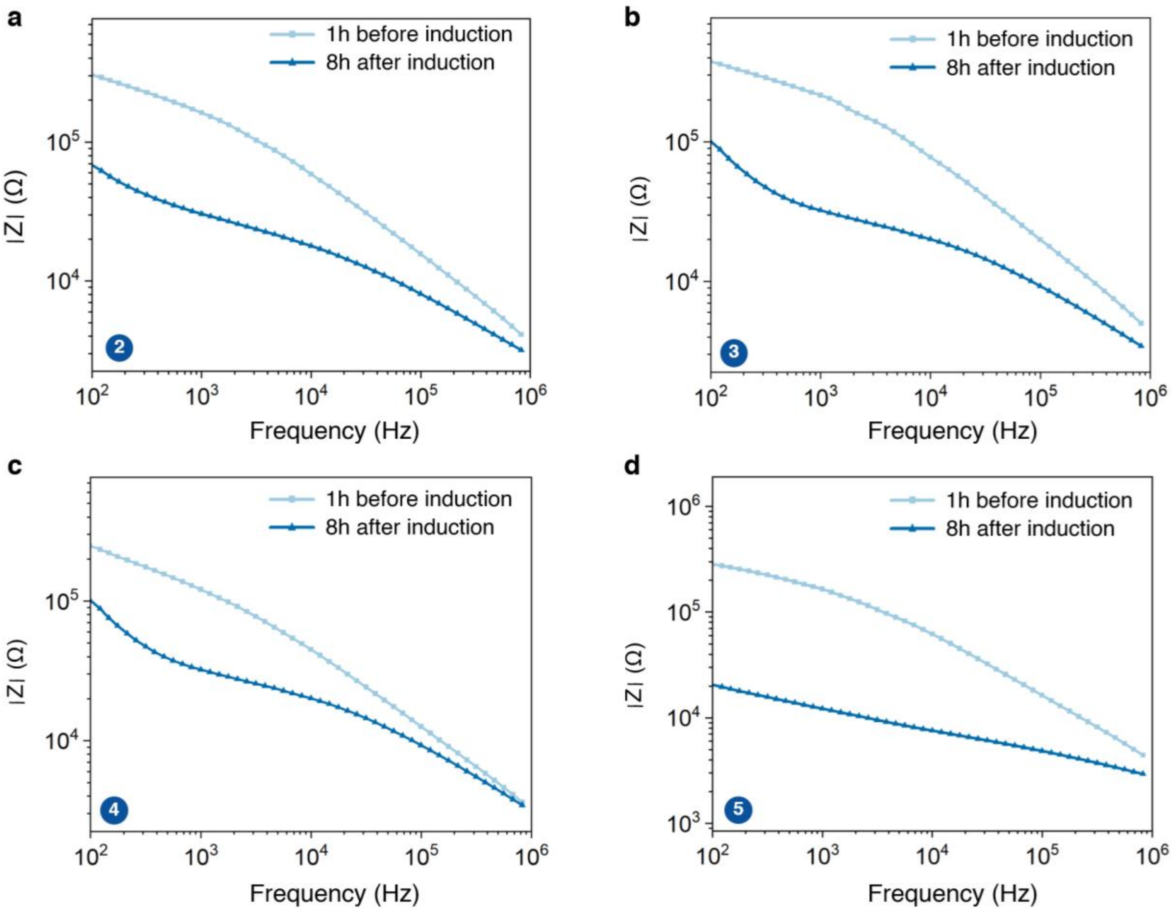

Supplementary Fig. 33. Impedance magnitude spectra of four different *A. thaliana* plants before and after induction with ethanol. a, Plant 2, **b,** Plant 3, **c**, Plant 4, **d,** Plant 5. Source data are provided as a Source Data file.

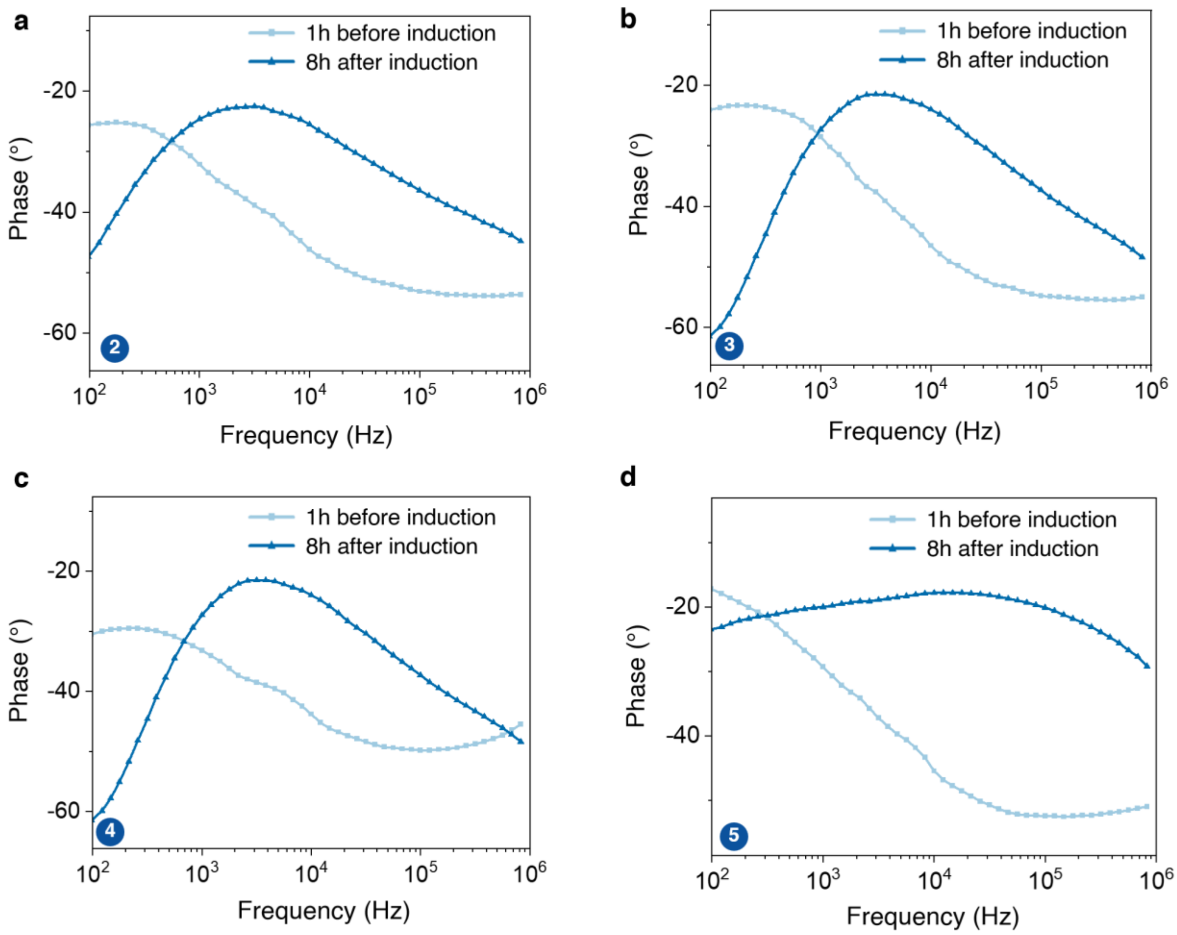

Supplementary Fig. 34. Phase spectra of four different *A. thaliana* plants before and after induction with ethanol. a, Plant 2, **b,** Plant 3, **c**, Plant 4, **d,** Plant 5. Source data are provided as a Source Data file.

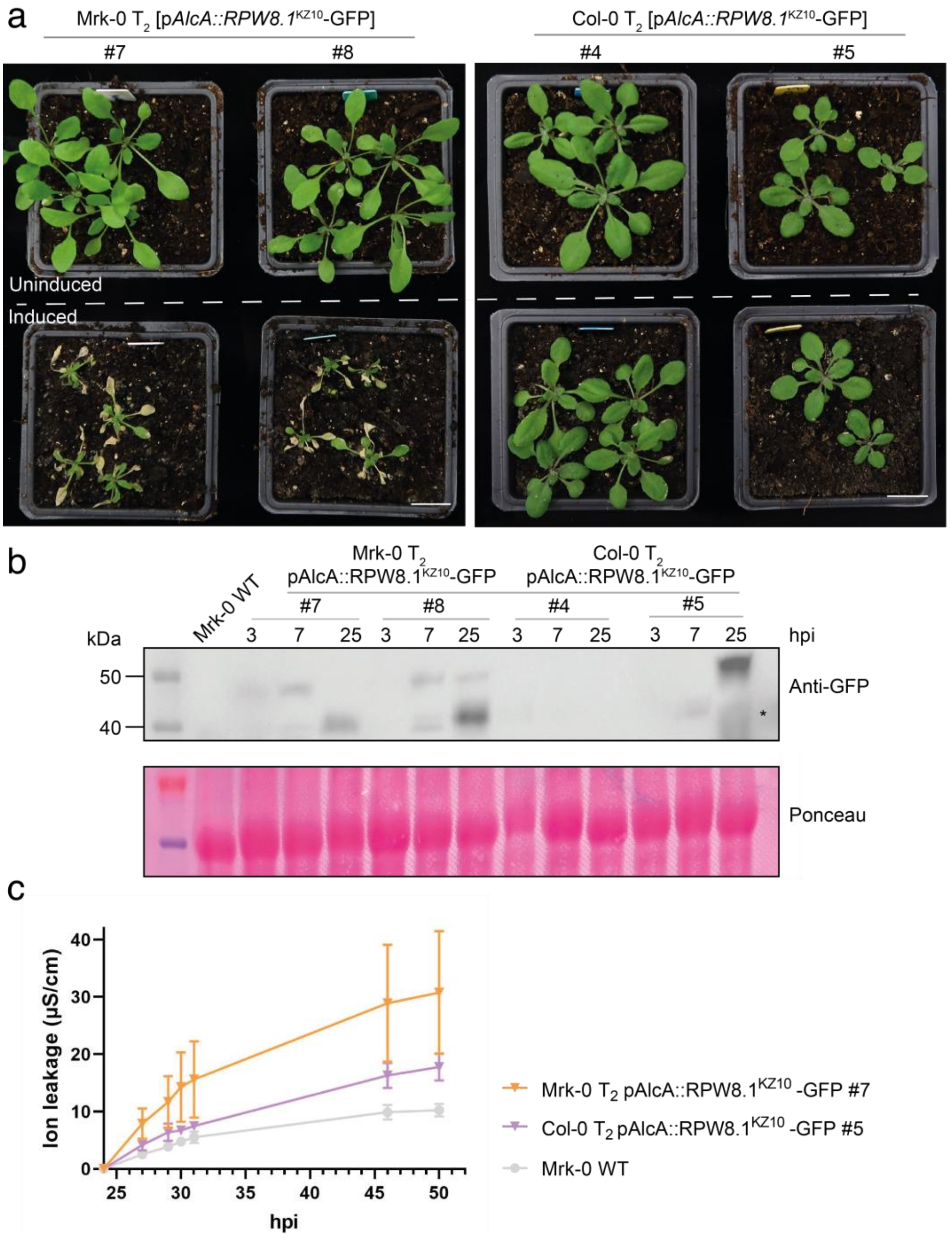

Supplementary Fig. 35. Ethanol-inducible RPW8.1^{KZ10}-GFP triggers cell death in Mrk-0 with paired NLR RPP7^{Mrk-0}. a, Phenotypes of T_2 transgenic Arabidopsis populations with ethanol-inducible RPW8.1^{KZ10}-GFP in Col-0 and Mrk-0 genetic backgrounds, observed 7 days post-induction with 1% ethanol. Photographs were taken of 30-day-old plants. Scale bar: 1 cm. **b**, Western blot analysis of total protein extracted from Arabidopsis leaves collected at the indicated time points (3 to 25 hours post-induction). The expression levels of GFP-tagged RPW8.1^{KZ10} were detected, and possible cleaved or degraded protein bands are marked with asterisks. Ponceau staining confirms comparable protein loading across samples. **c**, Ion leakage measurements as an indicator of cell death severity in the ethanol-inducible Mrk-0 and Col-0 transgenic two lines shown panel **a**. Source data are provided as a Source Data file.

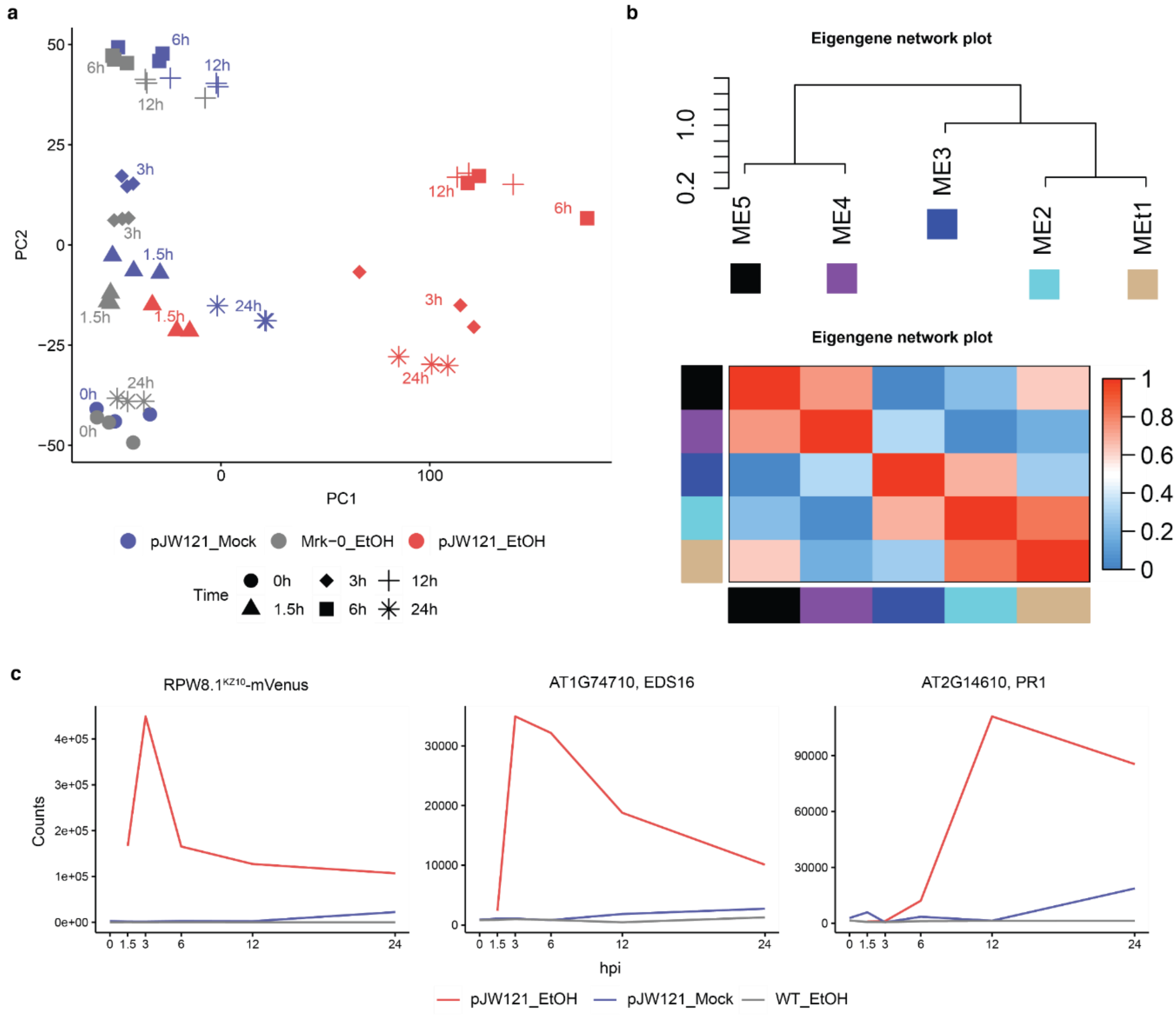

Supplementary Fig. 36. Principal component analysis (PCA) and eigengene network plot of inducible *DM6-DM7* time-course RNA-seq data in *A. thaliana*. a, PCA plot of transcriptomic data from Mrk-0_pJW121 (*pAlcA::RPW8.1*^{KZ10}*-mVenus*)_Mock and Mrk-0_pJW121 (*pAlcA::RPW8.1*^{KZ10}*-mVenus*)_EtOH treatments at various time points (0, 1.5, 3, 6, 12, 24 hours). Blue indicates mock-treated samples, red indicates ethanol-treated samples, and grey indicates ethanol-treated WT samples. PC1 accounts for 54% of the variance and PC2 accounts for 11%. **b,** Eigengene network plot from WGCNA displaying relationships between different modules (ME1-ME5). The dendrogram shows hierarchical clustering of module eigengenes, while the heatmap visualizes pairwise correlations between module eigengenes, with red indicating positive correlations and blue indicating negative correlations. **c,** Line plot of normalized counts from RNA-Seq, analyzed using DESeq2 with Wald test.

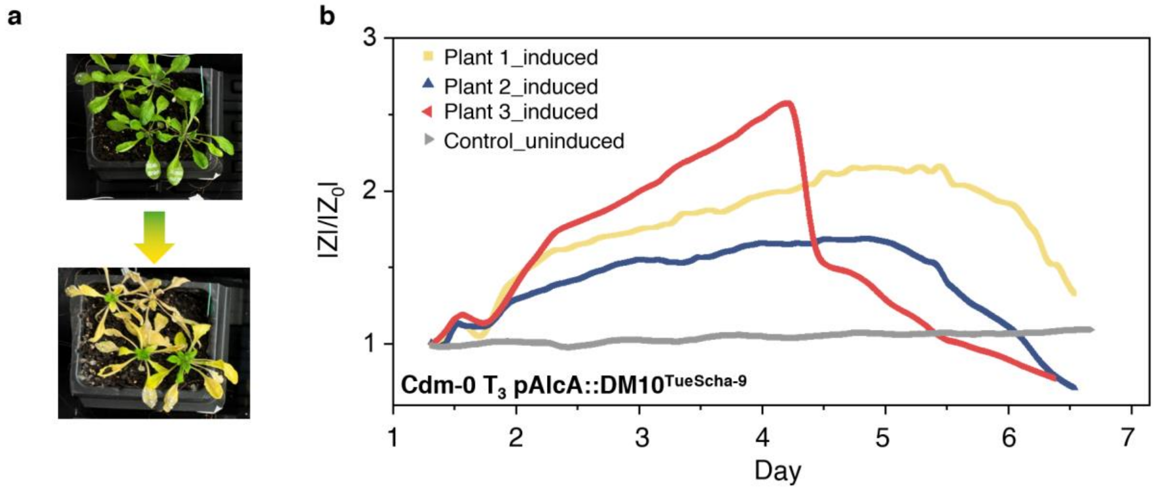

Supplementary Fig. 37. Monitoring TNL-mediated immune response in transgenic *A. thaliana* (Cdm-0) using a plant e-tattoo. a, Images of *A. thaliana* before and after ethanol induction to activate the DM10$^{\text{TueScha-9}}$ TNL[7] in the Cdm-0 background. Four-week-old transgenic Cdm-0 plants carrying *pAlcA::DM10*$^{\text{TueScha-9}}$ were treated with 1% ethanol via water irrigation for seven days under a covered dome. Tissue necrosis became visible after four days post-induction (dpi), with the lower image showing necrosis progression at 4 dpi. **b**, Normalized impedance magnitude at 2 kHz over time for the test and control groups following induction with ethanol or water. Source data are provided as a Source Data file.

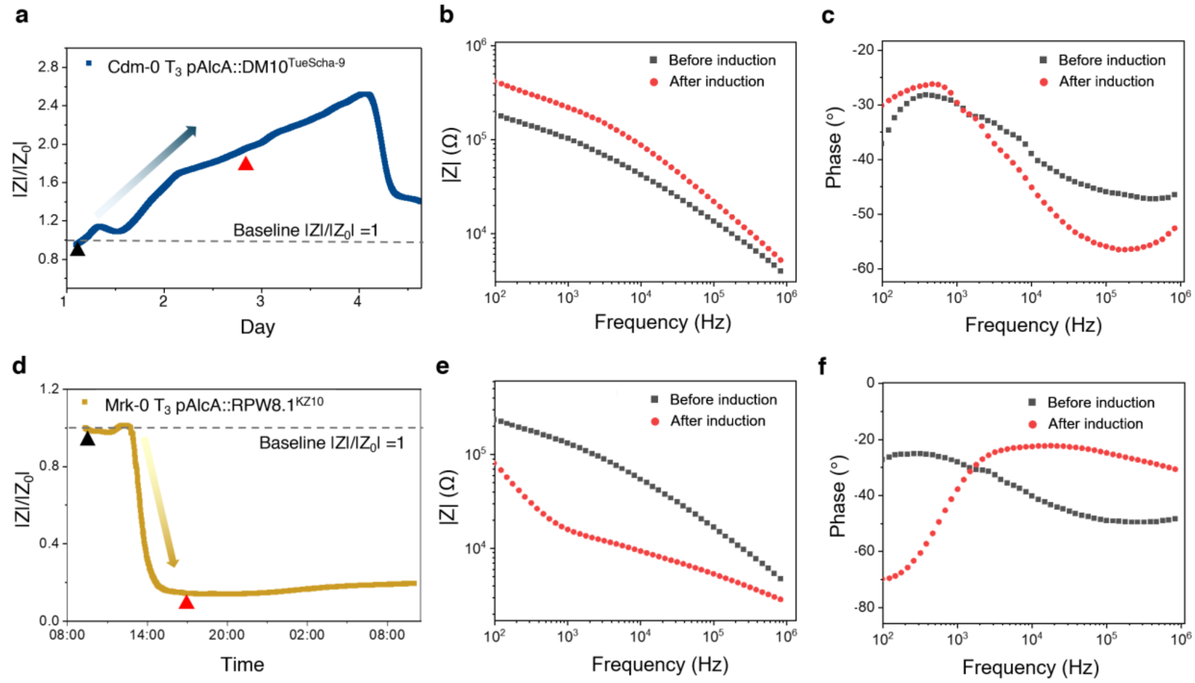

Supplementary Fig. 38. Impedance signature of CNL- and TNL- meditated immune response. a, Normalized impedance magnitude at 2 kHz of a typical *A. thaliana* (Cdm-0 T_3 *pAlcA::DM10*$^{\text{TueScha-9}}$) post induction with ethanol. **b-c**, The corresponding bode plots of the control and test plants collected at time points indicated with black and red triangular in **a**. **d,** Normalized impedance magnitude at 2 kHz of a typical *A. thaliana* (Mrk-0 T_3 *pAlcA::RPW8.1*$^{\text{KZ10}}$) post induction with ethanol. **e-f**, The corresponding bode plots of the control and test plants collected at time points indicated with black and red triangular in **d**. Source data are provided as a Source Data file.

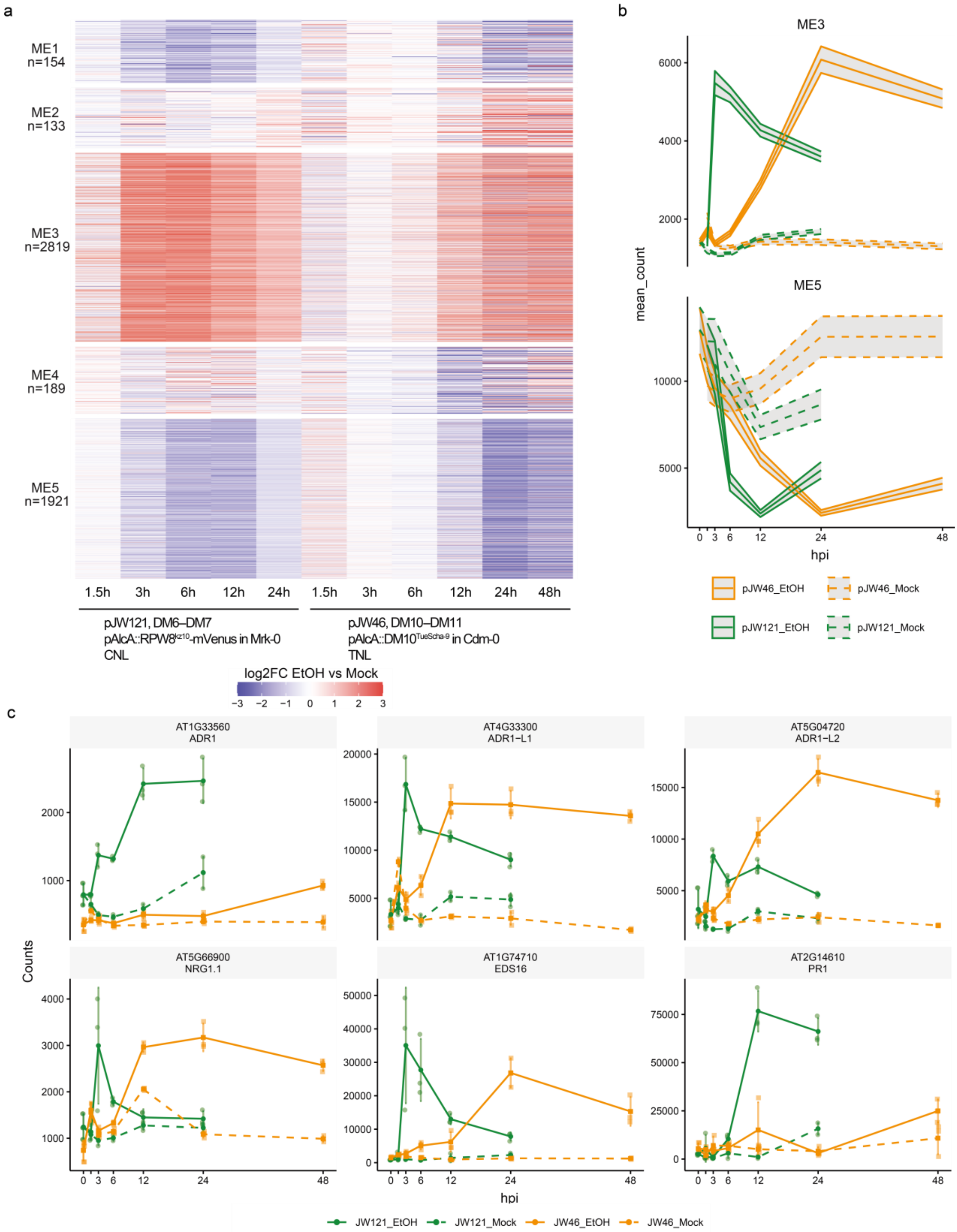

Supplementary Fig. 39. Comparative analysis of CNL and TNL-mediated transcriptional responses. a, Heatmap showing differential gene expression patterns (log2FC EtOH vs Mock) in CNL (*pAlcA::RPW8.1*$^{\mathrm{KZ10}}$*-mVenus*, *DM6-DM7*, pJW121) and TNL (*pAlcA::DM10*$^{\mathrm{TueScha\text{-}9}}$, *DM10-DM11*, pJW046) immune responses across indicated timepoints. Five major co-expression modules (ME1-ME5)

were identified through WGCNA analysis, with the number of genes in each module indicated. **b**, Temporal dynamics of ME3 (top) and ME5 (bottom) module eigengenes in CNL (*pAlcA::RPW8.1*^{KZ10}-*mVenus*, solid lines) and TNL (*pAlcA::DM10*$^{TueScha-9}$, dashed lines) immune responses. Shaded areas represent 95% confidence intervals calculated using Student's t-distribution (two-sided, n=3 biological replicates per condition). Orange and green lines represent pJW46 (*pAlcA::DM10*$^{TueScha-9}$) and pJW121 (*pAlcA::RPW8.1*^{KZ10}-*mVenus*) samples, respectively, with solid lines indicating EtOH treatment and dashed lines indicating mock treatment. **c**, Expression profiles of immune marker genes during CNL (*pAlcA::RPW8.1*^{KZ10}-*mVenus*, solid lines) and TNL (*pAlcA::DM10*$^{TueScha-9}$, dashed lines) activation. Selected genes include *ADR1* family members (AT1G33560, AT4G33300, AT5G04720), *NRG1.1* (AT5G66900), *EDS16* (AT1G74710), and *PR1* (AT2G14610). Data points represent mean ± standard error of the mean (SEM) from three biological replicates. Statistical differences between treatment groups were determined using DESeq2 with Wald test, with significance threshold set at adjusted p-value < 0.05. Orange and green lines indicate pJW46 and pJW121 samples respectively, with solid lines representing EtOH treatment and dashed lines representing mock treatment.

Supplementary Note 1. *DM6-DM7*-Mediated Autoimmunity in *Arabidopsis thaliana*.

The *DM6-DM7* NLR pair is a well-established genetic model for studying NLR-mediated autoimmunity. This autoimmunity occurs naturally in hybrid Arabidopsis accessions, where incompatible genetic interactions between *DM6* and *DM7* lead to spontaneous immune activation. QTL analysis has confirmed that the genomic regions encoding *DM6* and *DM7* are associated with hybrid necrosis in natural crosses, such as Lerik1-3 × Fei-0 and Mrk-0 × KZ10[8]. Mechanistic studies have demonstrated that *DM7*, a homolog of RPW8 ($HR4^{Fei-0}$), promotes the formation of a higher-order immune receptor complex involving $RPP7^{Lerik1-3}$ (*DM6*), a coiled-coil NLR (CNL). This complex assembly triggers downstream immune signaling, leading to cell death[9]. While DM7/RPW8 alone can induce a weak hypersensitive response (HR)—a form of programmed cell death associated with plant immunity—the simultaneous presence of both *DM6* and *DM7* results in a significantly stronger HR in both *A. thaliana* and *Nicotiana benthamiana*[9].

To validate that the observed autoimmunity is specifically mediated by NLR activity, we conducted a genetic experiment. We induced the expression of *DM7* $RPW8.1^{KZ10}$ in two distinct backgrounds: (1) Mrk-0, which carries the incompatible $RPP7^{Mrk-0}$ partner, and (2) Col-0, which lacks this NLR partner. Notably, cell death was observed only in the Mrk-0 transgenic lines, demonstrating that the presence of RPP7 is essential for triggering autoimmunity. This finding provides strong evidence that the immune response we detected is driven by an NLR-specific signaling mechanism rather than non-specific stress responses or other immune pathways.

Phenotypic observations revealed that cell death was visible only in Mrk-0 transgenic lines following induction, while Col-0 remained unaffected (Supplementary Fig. 35a). Western blot analysis confirmed the successful induction of *DM7* expression in both genetic backgrounds, as evidenced by the presence of GFP-tagged $RPW8.1^{KZ10}$ (Supplementary Fig. 35b). Despite comparable expression levels, only the Mrk-0 transgenic lines exhibited a robust hypersensitive response, reinforcing the necessity of RPP7 for a strong immune reaction. Additionally, ion conductivity measurements provided quantitative evidence of cell membrane damage, a hallmark of cell death. Conductivity levels increased rapidly and significantly in the Mrk-0 transgenic lines, reflecting severe membrane disruption. In contrast, Col-0 displayed a slower and milder increase in conductivity, consistent with the weaker response expected in the absence of RPP7 (Supplementary Fig. 35c). These observations align with previous findings reported by Li *et al.* [9], further validating the role of the *DM6-DM7* pair functions as an NLR-based immune signaling module responsible for triggering autoimmunity.

Supplementary Note 2. TNL-mediated immune responses monitoring.

Plant immunity relies on intracellular nucleotide-binding leucine-rich repeat (NLR) receptors, which are categorized into two primary classes: CNLs (coiled-coil NLRs) and TNLs (Toll/interleukin-1 receptor-like NLRs). These receptors detect pathogen-derived effectors and trigger defense responses, often culminating in a localized hypersensitive response (HR) to contain the infection. While both CNLs and TNLs contribute to robust immune signaling, they exhibit distinct structural and functional characteristics.

CNLs, characterized by their coiled-coil (CC) domains, are prevalent across plant species and are known for their rapid immune activation upon pathogen detection. A prominent example is ZAR1, a CNL in *A. thaliana*, which oligomerizes into a pentameric resistosome upon activation. The ZAR1 resistosome functions as a calcium-permeable channel, facilitating the influx of calcium ions—a critical second messenger in plant immune signaling. This rapid calcium signaling underpins the swift activation of defense responses by CNLs, allowing for immediate containment of pathogen attacks[10]. In contrast, TNLs, defined by their Toll/interleukin-1 receptor (TIR) domains, are predominantly found in dicots and rely on more complex signaling pathways. TNL activation involves helper proteins such as EDS1 and SAG101, which mediate transcriptional reprogramming and amplify immune responses. Compared to the direct signaling of CNLs, TNL-mediated immunity is slower, reflecting a more layered and nuanced defense strategy. This differentiation in response speed and complexity highlights the complementary roles of CNLs and TNLs in plant immunity[11,12].

The mechanistic diversity between CNL- and TNL-mediated immunity provides a foundation for studying their distinct signaling pathways and physiological impacts. By leveraging phenotypic tools such as plant e-tattoo, it is possible to monitor immune activation and associated cell death dynamics with high specificity, enabling deeper insights into plant immune mechanisms.

We have established the *DANGEROUS MIX* (*DM*) autoimmunity system, which induces a purely NLR-mediated immune response without requiring pathogen treatment[13]. This system provides a well-defined and controllable platform for specifically investigating NLR-mediated immunity, eliminating confounding factors associated with other immune pathways. To ensure precise control over immune activation, we established stable *A. thaliana* transgenic lines that allow genetic induction of NLR-mediated responses with minimal external intervention. Using an ethanol-inducible promoter (*pAlcA*), we selectively activated one incompatible, namely *DANGEROUS MIX* (*DM*) partner, enabling precise temporal control over NLR-triggered immunity. Specifically, we generated two ethanol-inducible lines:

- ***DM6-DM7* case:** Stable transgenic lines carrying *pAlcA::RPW8.1*$^{\mathrm{KZ10}}$ in Mrk-0 accession background, which activates CNL-mediated immune responses
- ***DM10-DM11* case:** Stable transgenic lines carrying *pAlcA::DM10*$^{\mathrm{TueScha\text{-}9}}$ in Cdm-0 accession background, which activates TNL-mediated immune response

Due to the response kinetics, our study primarily focuses on CNL (coiled-coil NLR)-mediated immune responses, specifically the *DM6-DM7* case. We have provided substantial evidence demonstrating the feasibility of using the e-tattoo to monitor this type of NLR-mediated response (Fig. 5, 6, Supplementary Fig. 23-36). Additionally, we have included data (Supplementary Fig. 37,38) showing that the e-tattoo

successfully detects impedance changes associated with TNL (Toll/interleukin-1 receptor-like NLR)-mediated immune activation in the Cdm-0 T_3 [*pAlcA::DM10*$^{TueScha-9}$] line (*DM10-DM11* case)[7].

The *DM10-DM11* system serves as a robust model for investigating Toll/interleukin-1 receptor-like nucleotide-binding leucine-rich repeat (TNL) receptor-mediated immune responses in *A.thaliana*. *DM10-DM11* autoimmunity is triggered by the co-occurrence of two specific TNL genes: *DM10*, identified in the natural accession TueScha-9, and the yet-to-be-identified *DM11*, originating from Cdm-0. Together, these genes encode components that assemble into a functional immune complex, providing a controlled framework for studying TNL-mediated immunity[7].

TNL- and CNL-mediated immune responses exhibit distinct signaling mechanisms and kinetics, resulting in different impedance signatures. CNL-mediated immune responses, such as those triggered by AvrRpt2[14], induce rapid HR and cell death. Recent studies have demonstrated that ZAR1, a well-characterized CNL, forms a calcium-permeable resistosome, leading to a swift ion influx and rapid HR within hours post-activation[10]. This provides mechanistic support for the rapid impedance decrease we observed. In contrast, TNL-mediated responses, such as those triggered by AvrRps4, involve a slower yet prolonged activation phase, requiring downstream components like EDS1 and NRG1[14]. The extended kinetics of TNL responses align with our impedance measurements and reinforce the conceptual distinction between these two immune receptor classes. For CNL-mediated immune responses, we observed a sharp decrease in impedance magnitude within ~3 hours post-induction. In contrast, TNL activation displayed a different pattern: impedance magnitude gradually increases, reaching a peak 4-6 dpi, followed by a sharp decline, indicative of severe cell death. This prolonged response reflects the slower propagation of TNL-mediated immunity compared to CNL.

To further validate the specificity of our e-tattoo in detecting distinct NLR-mediated immune responses, we performed comparative transcriptome analysis between CNL and TNL-activated immunity. WGCNA analysis identified five major co-expression modules (ME1-ME5) that exhibited distinct temporal patterns between CNL and TNL activation (Supplementary Fig. 39a). Notably, ME3 and ME5, which represent major immune response signatures, showed contrasting dynamics between CNL and TNL activation (Supplementary Fig. 39b). ME3, enriched in early immune response genes, showed rapid induction in CNL-mediated immunity within 3-6 hours, while displaying delayed activation in TNL-mediated responses. Conversely, ME5 exhibited sustained suppression in CNL responses but gradual recovery in TNL responses. These distinct module patterns were further reflected in the expression profiles of key immune marker genes (Supplementary Fig. 39c). CNL activation triggered rapid induction of defense-related genes, with expression peaks occurring within 3-6 hours post-induction, while TNL-mediated responses showed delayed but sustained activation peaking between 12-24 hours. These distinct transcriptional kinetics align with our impedance measurements, demonstrating that our e-tattoo can effectively distinguish between different NLR-mediated immune responses, thereby validating its specificity and utility for monitoring distinct immune activation patterns in plants.

Compared to traditional pathogen-based approaches, our genetically controlled model offers a more precise and reproducible method for studying NLR-mediated immunity, ensuring that observed impedance changes can be attributed directly to NLR activation. Thus, our current study establishes a rigorous and controlled framework for characterizing NLR-specific impedance signatures, providing a solid foundation for future investigations into more diversified stress responses.

Supplementary references

1 Bukhamsin, A. *et al.* Robust, long-term, and exceptionally sensitive microneedle-based bioimpedance sensor for precision farming. *Advanced Science* **8**, 2101261 (2021).

2 Kim, J. J., Allison, L. K. & Andrew, T. L. Vapor-printed polymer electrodes for long-term, on-demand health monitoring. *Science Advances* **5**, eaaw0463 (2019).

3 Kim, J. J., Fan, R., Allison, L. K. & Andrew, T. L. On-site identification of ozone damage in fruiting plants using vapor-deposited conducting polymer tattoos. *Science Advances* **6**, eabc3296 (2020).

4 Barbosa, J. A. *et al.* Biocompatible wearable electrodes on leaves toward the on-site monitoring of water loss from plants. *ACS Applied Materials & Interfaces* **14**, 22989-23001 (2022).

5 Qu, C., Cao, L., Li, M., Wang, X. & He, Z. Liquid metal-based plant electronic tattoos for in-situ monitoring of plant physiology. *Science China Technological Sciences* **66**, 1617-1628 (2023).

6 Peng, B. *et al.* In-time detection of plant water status change by self-adhesive, water-proof, and gas-permeable electrodes. *ACS Applied Materials & Interfaces* **15**, 19199-19208 (2023).

7 Barragan, A. C. *et al.* A truncated singleton NLR causes hybrid necrosis in *Arabidopsis thaliana*. *Molecular Biology and Evolution* **38**, 557-574 (2021).

8 Barragan, C. A. *et al.* RPW8/HR repeats control NLR activation in *Arabidopsis thaliana*. *PLoS Genetics* **15**, e1008313 (2019).

9 Li, L., Habring, A., Wang, K. & Weigel, D. Atypical resistance protein RPW8/HR triggers oligomerization of the NLR immune receptor RPP7 and autoimmunity. *Cell Host & Microbe* **27**, 405-417. e406 (2020).

10 Bi, G. *et al.* The ZAR1 resistosome is a calcium-permeable channel triggering plant immune signaling. *Cell* **184**, 3528-3541. e3512 (2021).

11 Kourelis, J. & Van Der Hoorn, R. A. Defended to the nines: 25 years of resistance gene cloning identifies nine mechanisms for R protein function. *Plant Cell* **30**, 285-299 (2018).

12 Jones, J. D., Staskawicz, B. J. & Dangl, J. L. The plant immune system: From discovery to deployment. *Cell* **187**, 2095-2116 (2024).

13 Yang, Y., Duan, S. & Zhao, H. Advances in constructing silver nanowire-based conductive pathways for flexible and stretchable electronics. *Nanoscale* **14**, 11484-11511 (2022).

14 Saile, S. C. *et al.* Two unequally redundant" helper" immune receptor families mediate Arabidopsis thaliana intracellular" sensor" immune receptor functions. *PLoS Biology* **18**, e3000783 (2020).
