# Supplementary material for: Epidermal electronic-tattoo for plant immune response monitoring
Source: Nat Commun. 2025 Apr 4;16:3244. doi: 10.1038/s41467-025-58584-x (PMC11971386; doi:10.1038/s41467-025-58584-x)
Supplement: Supplementary file 3 — Description of Additional Supplementary Files [file 41467_2025_58584_MOESM3_ESM.pdf]

Description of Additional Supplementary Files

File Name: Supplementary Movie 1
Description: **Non-invasive in-water transfer printing process.** The plant epidermal etattoo, made of a substrate-free AgNW network, is fabricated using a vacuum filtration process. It can be released onto the water surface and gently transferred to the leaf surface, ensuring a safe application.
