# Supplementary material for: Epidermal electronic-tattoo for plant immune response monitoring
Source: Nat Commun. 2025 Apr 4;16:3244. doi: 10.1038/s41467-025-58584-x (PMC11971386; doi:10.1038/s41467-025-58584-x)
Supplement: Supplementary file 5 — Reporting Summary [file 41467_2025_58584_MOESM5_ESM.pdf]

nature portfolio

Corresponding author(s): Chengkuo Lee, Eunyoung Chae

Last updated by author(s): Mar 17, 2025

Reporting Summary

Nature Portfolio wishes to improve the reproducibility of the work that we publish. This form provides structure for consistency and transparency in reporting. For further information on Nature Portfolio policies, see our Editorial Policies and the Editorial Policy Checklist.

Statistics

For all statistical analyses, confirm that the following items are present in the figure legend, table legend, main text, or Methods section.

n/a	Confirmed	
☐	☒	The exact sample size (*n*) for each experimental group/condition, given as a discrete number and unit of measurement
☐	☒	A statement on whether measurements were taken from distinct samples or whether the same sample was measured repeatedly
☐	☒	The statistical test(s) used AND whether they are one- or two-sided<br>*Only common tests should be described solely by name; describe more complex techniques in the Methods section.*
☐	☒	A description of all covariates tested
☐	☒	A description of any assumptions or corrections, such as tests of normality and adjustment for multiple comparisons
☐	☒	A full description of the statistical parameters including central tendency (e.g. means) or other basic estimates (e.g. regression coefficient) AND variation (e.g. standard deviation) or associated estimates of uncertainty (e.g. confidence intervals)
☐	☒	For null hypothesis testing, the test statistic (e.g. *F*, *t*, *r*) with confidence intervals, effect sizes, degrees of freedom and *P* value noted<br>*Give P values as exact values whenever suitable.*
☒	☐	For Bayesian analysis, information on the choice of priors and Markov chain Monte Carlo settings
☒	☐	For hierarchical and complex designs, identification of the appropriate level for tests and full reporting of outcomes
☒	☐	Estimates of effect sizes (e.g. Cohen's *d*, Pearson's *r*), indicating how they were calculated

Our web collection on statistics for biologists contains articles on many of the points above.

Software and code

Policy information about availability of computer code

Data collection	The RNA-seq data was generated using standard Illumina sequencing protocols on the NovaSeq X Plus platform. Arduino Software v1.8.13 (IDE) was used for continuous impedance recording. No custom software was used for data collection.
Data analysis	RNA-seq data analysis was performed using standard bioinformatics tools and R packages. Read mapping was performed using Hisat2. Read counting was done using featureCounts from the Subread package. Differential expression analysis was conducted using DESeq2 in R. Weighted gene co-expression network analysis was performed using WGCNA package in R. Data visualization was created using ggplot2. All analyses used default parameters unless otherwise specified in the Methods section.

For manuscripts utilizing custom algorithms or software that are central to the research but not yet described in published literature, software must be made available to editors and reviewers. We strongly encourage code deposition in a community repository (e.g. GitHub). See the Nature Portfolio guidelines for submitting code & software for further information.

Data

Policy information about availability of data

All manuscripts must include a data availability statement. This statement should provide the following information, where applicable:

- Accession codes, unique identifiers, or web links for publicly available datasets
- A description of any restrictions on data availability
- For clinical datasets or third party data, please ensure that the statement adheres to our policy

The RNA-Seq data generated in this study has been deposited in NCBI under BioProject accession number PRJNA1080050. The authors declare that all other data

supporting the findings of this study are available within the paper and its Supplementary Information. Any additional requests for information can be directed to, and will be fulfilled by, the lead contact. Source data are provided with this paper.

Research involving human participants, their data, or biological material

Policy information about studies with human participants or human data. See also policy information about sex, gender (identity/presentation), and sexual orientation and race, ethnicity and racism.

Reporting on sex and gender	N/A
Reporting on race, ethnicity, or other socially relevant groupings	N/A
Population characteristics	N/A
Recruitment	N/A
Ethics oversight	N/A

Note that full information on the approval of the study protocol must also be provided in the manuscript.

Field-specific reporting

Please select the one below that is the best fit for your research. If you are not sure, read the appropriate sections before making your selection.

☒ Life sciences ☐ Behavioural & social sciences ☐ Ecological, evolutionary & environmental sciences

For a reference copy of the document with all sections, see nature.com/documents/nr-reporting-summary-flat.pdf

Life sciences study design

All studies must disclose on these points even when the disclosure is negative.

Sample size	Sample size was determined based on standard practices in plant transcriptomics research. For RNA-Seq analysis, we used three biological replicates per time point per treatment group, with each replicate containing tissue from at least 3 individual plants to account for plant-to-plant variation. This sample size provides sufficient statistical power to detect differentially expressed genes while balancing experimental feasibility.
Data exclusions	No data were excluded from the RNA-Seq analyses. All sequenced samples met quality control standards and were included in the final analysis.
Replication	RNA-Seq experiments were conducted with three independent biological replicates per time point per treatment condition. All findings were successfully replicated across these biological replicates. The gene expression patterns and module identification through WGCNA were consistent across replicates.
Randomization	Plants were randomly assigned to treatment groups (ethanol vs. mock treatment). During the growth period, plant positions were randomized and pots were re-arranged every other day within the growth chamber to minimize potential position effects due to light intensity variation. This randomization strategy was maintained throughout the experiment until sample collection.
Blinding	Complete blinding was not possible during treatment and sample collection due to the nature of ethanol treatment, which required separate chamber setup and produced distinct phenotypes and odors. However, subsequent RNA extraction, library preparation, and bioinformatic analyses were conducted without knowledge of sample identity to minimize potential bias. Sample labels were coded during sequencing and initial data processing steps to maintain objectivity during analysis.

Reporting for specific materials, systems and methods

We require information from authors about some types of materials, experimental systems and methods used in many studies. Here, indicate whether each material, system or method listed is relevant to your study. If you are not sure if a list item applies to your research, read the appropriate section before selecting a response.

Materials & experimental systems

n/a	Involved in the study
☐	☒ Antibodies
☒	☐ Eukaryotic cell lines
☒	☐ Palaeontology and archaeology
☒	☐ Animals and other organisms
☒	☐ Clinical data
☒	☐ Dual use research of concern
☐	☒ Plants

Methods

n/a	Involved in the study
☒	☐ ChIP-seq
☒	☐ Flow cytometry
☒	☐ MRI-based neuroimaging

Antibodies

Antibodies used	A single antibody was utilized in this study. The GFP-HRP antibody, sourced from Miltenyi Biotec, was employed to detect RPW8-mVenus/GFP protein expression levels. It is identified by the clone name GG4-2C2.12.10, with catalog number 130-091-833 and lot number 5240303175.
Validation	According to the manufacturer's website (https://www.miltenyibiotec.com/SG-en/products/gfp-antibody-gg4-2c2-12-10.html), this antibody can be used for antigen (GFP) in any cell type for western blot.

Plants

Seed stocks	Arabidopsis thaliana accession Col-0 (ABRC stock number: CS70000) Cdm-0 (ABRC stock number: CS76410) and Mrk-0 seeds (ABRC stock number: CS1374) are from lab stock.
Novel plant genotypes	Col-0 and Mrk-0 plants carrying the inducible DM6-DM7 transgene were generated using the floral dipping method. The specific lines include Mrk-0 T3[pAlcA::RPW8.1KZ10-mVenus] #1, Col-0 T2[pAlcA::RPW8.1KZ10-GFP] #4 and #5, Mrk-0 T2[pAlcA::RPW8.1KZ10-GFP] #7 and #8 and Cdm-0 T3[pAlcA::DM10TueScha-9] #2 and #5. Three independent lines were analyzed, and those exhibiting the typical phenotype were selected for this study. The second (T2) and third (T3) generations were used in the research.
Authentication	The transgenic lines were selected using basta (DM10), kanamycin and hygromycin B (RPW8) on 1/2 MS agar plates. Non-transgenic plants, Col-0 WT, Cdm-0 WT and Mrk-0 WT, were grown alongside transgenic Col-0 and Mrk-0 T2/3[pAlcA::RPW8.1KZ10-mVenus/GFP] and Cdm-0 T3[pAlcA::DM10TueScha-9] plants to evaluate whether, in the absence of ethanol induction of RPW8.1 and DM10, the transgenic lines exhibited a phenotype comparable to Col-0 WT, Mrk-0 and Cdm-0. The transgenic lines displayed a similar phenotype to the WT lines under these conditions, indicating that either pAlcA::RPW8 and pAlcA::DM10 transgene had no observable side effects.
